# Dengue Virus Capsid Interacts with DDX3X–A Potential Mechanism for Suppression of Antiviral Functions in Dengue Infection

**DOI:** 10.3389/fcimb.2017.00542

**Published:** 2018-01-17

**Authors:** Rinki Kumar, Nirpendra Singh, Malik Z. Abdin, Arvind H. Patel, Guruprasad R. Medigeshi

**Affiliations:** ^1^Clinical and Cellular Virology Lab, Vaccine and Infectious Disease Research Center, Translational Health Science and Technology Institute, NCR-Biotech Science Cluster, Faridabad, India; ^2^Department of Biotechnology, Jamia Hamdard, New Delhi, India; ^3^Regional Center for Biotechnology, NCR-Biotech Science Cluster, Faridabad, India; ^4^MRC-University of Glasgow Centre for Virus Research, Glasgow, United Kingdom

**Keywords:** Dengue virus, capsid, DDX3X, siRNA, antiviral

## Abstract

Dengue virus is a pathogen of global concern and has a huge impact on public health system in low- and middle-income countries. The capsid protein of dengue virus is least conserved among related flavivirus and there is very limited information on the role of cytosolic proteins that interact with dengue virus capsid. We identified DEAD (Asp-Glu-Ala-Asp) Box Helicase 3, an X-Linked (DDX3X), cytosolic ATP-dependent RNA helicase as a dengue virus capsid-interacting protein. We show that the N-terminal region of capsid is important for interaction with DDX3X, while the N-terminal domain of DDX3X seems to be involved in interaction with dengue capsid. DDX3X was down-regulated in dengue virus infected cells at later stages of infection. Our results show that DDX3X is an antiviral protein as suppression of DDX3X expression by siRNA led to an increase in viral titers and overexpression of DDX3X led to inhibition of viral replication. Knock-down of DDX3X did not affect induction of type I interferon response upon infection suggesting that the effect of DDX3X knock-down is independent of the interferon-dependent pathways that DDX3X modulates under normal conditions. Thus, our study identifies DDX3X as a dengue virus capsid interacting protein and indicates a potential link between the antiviral functions of DDX3X and dengue capsid at later stages of dengue infection.

## Introduction

Dengue is the most common mosquito-borne viral disease in tropical and subtropical areas, with an estimated 50–100 million infections occurring each year. Dengue virus (DENV) is a member of *Flaviviridae* family, which consists of a group of enveloped, positive sense, single-stranded, RNA viruses. DENV genome encodes three structural proteins, namely core (C), membrane (prM/M), and envelope (E), and seven non-structural proteins, (NS1, NS2A, NS2B, NS3, NS4A, NS4B, and NS5), which are produced via proteolytic processing of the single polyprotein by viral serine protease (NS2B-NS3) and cellular proteases (Medigeshi, 2011). Sequential cleavages of the anchored capsid protein by viral protease and host signalase result in removal of the C-terminal trans-membrane region to yield the 100-residue mature form, which is a highly basic 12 kDa protein (Amberg et al., 1994; Yamshchikov and Compans, 1995; Sangiambut et al., 2008, 2013). While the mechanism by which encapsidation occurs is not completely understood, it is proposed that the capsid protein interacts with the viral genome to form the nucleocapsid, possibly via interactions with basic surfaces on the solvent-exposed side of the protein (Jones et al., 2003; Ma et al., 2004). A number of host proteins have been identified as capsid-interacting proteins for various flaviviruses and these studies implicate capsid in a variety of functions such as lipid metabolism, apoptosis and stress granule formation (Byk and Gamarnik, 2016). West Nile virus capsid protein has been proposed to activate pro-survival pathways by blocking apoptosis through a phosphatidylinositol (PI) 3-kinase-dependent pathway (Urbanowski and Hobman, 2013). However, another study showed an opposite role for capsid where, WNV capsid was shown to induce apoptosis in a p53-dependent manner (Yang et al., 2008). Recent studies have also proposed a role for JEV capsid protein in blocking stress granule formation and relieving translation repression by interaction with Caprin-1 (Katoh et al., 2013). DENV capsid was shown to colocalize with MX1/MxA, an interferon-induced GTPase involved in antiviral signaling in primary endothelial cells infected with DENV-2 suggesting a role for capsid protien in blocking antiviral pathways (Kanlaya et al., 2010).

Many groups have identified capsid-interacting proteins some of which include nucleolin, histone proteins H2A, H2B, H3, H4, and importin-α (Bhuvanakantham et al., 2009; Netsawang et al., 2010; Colpitts et al., 2011; Balinsky et al., 2013; Bhuvanakantham and Ng, 2013; Mairiang et al., 2013). Overexpression of capsid protein leads to nuclear localization, therefore, it is not surprising, that most of the capsid-interacting proteins identified above are nuclear proteins. Although the C protein of dengue virus localizes to the nucleus of mammalian cells and remains at high levels throughout the course of infection (Wang et al., 2002; Sangiambut et al., 2008), its role in the nucleus is not yet understood. Given the role of C in promoting encapsidation of the viral RNA and subsequent assembly of infectious virus particles, it is imperative to identify non-nuclear capsid-interacting proteins to further elucidate the role of capsid in dengue virus life-cycle. In this study, we adopted an in-solution interaction approach and identified 16 proteins interacting with DENV capsid by proteomics and mass spectrometry. One of the capsid-interacting proteins identified was DDX3X, a DEAD (Asp-Glu-Ala-Asp)-box helicase. DEAD-box RNA helicases belong to helicase superfamily 2 and consist of two large domains, the N- terminal and C-terminal domains which consist of 9 conserved sequence motifs (Garbelli et al., 2011b). DDX3X shuttles between cytoplasm and the nucleus and plays a multifunctional role such as RNA splicing, transcriptional and translational regulation, mRNA export and translation initiation, and it also contributes to the nuclear export of RNA (Owsianka and Patel, 1999; Yedavalli et al., 2004; Lai et al., 2008; Schroder, 2011). DDX3X has also been shown to regulate innate immune responses through its interaction with components of antiviral signaling such as mitochondrial antiviral signaling protein, TANK-binding kinase-1 and I-kappa-B kinase epsilon which results in IRF3 and IRF7 activation and interferon-β production (Schröder et al., 2008; Soulat et al., 2008; Mulhern and Bowie, 2010; Oshiumi et al., 2010b). Recent reports suggests that many viruses usurp the function of DDX3X to promote viral RNA replication or prevent DDX3X functions (Valiente-Echeverría et al., 2015). Therefore, we further characterized the role of DDX3X in dengue infection as a capsid-interacting partner. We show that DDX3X is an antiviral protein and interaction of DENV capsid with DDX3X may counter the antagonistic effect of DDX3X in DENV life-cycle.

## Materials and methods

### Cells and viruses

All the cell lines, virus strain used in this study and virus titering protocols have been described previously (Agrawal et al., 2013; Medigeshi et al., 2016). Huh-7, HEK293T, A549 cells were grown in Dulbecco's modified Eagle medium (DMEM) supplemented with 10% fetal bovine serum (FBS), 1 mM L-glutamine, 100 units/ml penicillin-streptomycin-glutamine and non-essential amino acids at 37°C and 5% CO2. BHK-21 and C6/36 cells were grown in the minimal essential medium (MEM) containing 10% FBS, Earl's salts and PSG in the concentration mentioned above. Dengue virus serotype 2 obtained from National Institute of virology, India (Dengue virus 2 isolate P23085 INDI-60- GenBank: KJ918750.1) was used throughout the study. The virus was cultured in C6/36 cell lines and titered by plaque assay in BHK21 cells as described previously (Agrawal et al., 2013).

### Antibodies

Mouse monoclonal antibodies for anti-6X his-tag (Abcam - ab18184) (1:1,000), HA (Biolegend 901501) (1:1,000), α-tubulin (Clone 12G10 from Developmental Studies Hybridoma Bank, University of Iowa) (1:5,000) and viral envelope (Merck-Millipore MAB10216) (1:1,000) were used. Rabbit polyclonal antibodies for GAPDH (1:5,000) were from Cell Signaling Technology (CST 2118). Rabbit polyclonal dengue-capsid antibody was used for immunoblotting (1:1,000) (Agrawal et al., 2013). Rabbit polyclonal DDX3X (R648) antibody (1:2,000 for western blotting and 1:1,000 for immunofluorescence) has been described before (Angus et al., 2010). Anti-rabbit (Merck-Millipore) or anti-mouse (Sigma) IgG secondary antibodies raised in goat and conjugated with horseradish peroxidase (HRP) (1:2,000) were used. HRP-conjugated secondary antibodies specific to IgG-light chain (Jackson's Immunoresearch Lab) were used (1:10,000) for probing blots of immunoprecipitation.

### Cloning, expression, and purification of DENV2 capsid

DENV2 full-length capsid construct was generated by PCR and cloning into pET-23b vector using the forward primer 5′-TACATATGAATAACCAACGGAAAAAGG-3′ and reverse primer 5′-GACTCGAGTCTGCGTCTCCTGTTCAAG-3′ using NdeI & XhoI sites. Capsid clone in a mammalian expression vector pEF1/*myc*hisC was generated using the forward primer 5′-TAGGTACCATGAATAACCAACGGAAAAAG-3′ and reverse primer 5′-TAGCGGCCGCTCAGTGGTGGTGGTGGTG-3′ with KpnI and NotI restriction sites.

Capsid deletion constructs were generated in pEF1/*myc*hisA with KpnI & NotI sites using the following primers: α2-4-capsid (26-100 a.a) 5′-TAGGTACCATGAAACTGTTCATGGCCC-3′ (forward) and 5′-TAGCGGCCGCTCAGTGGTGGTGGTGGTG-3′ (reverse). α3-4-capsid (63–100 a.a) 5′-TAGGTACCATGGCAGGGATATTGAAGA-3′(forward) and 5′ TAGCGGCCGCTCAGTGGTGGTGGTGGTG-3′ (reverse). α4-capsid (74–100 a.a) 5′-TAGGTACCATGAAATCAAAAGC-3′ (forward) and 5′-TAGCGGCCGCTCAGTGGTGGTGGTGGTG-3′ (reverse). Plasmids were constructed by standard PCR methods using Expand Hi fidelity^plus^ PCR system (Roche). Clones were verified by sequencing.

### Protein purification

The His-tagged full length DENV2 capsid clone was generated in pET23b vector as described above, and expressed in BL21DE3pLysS cells for protein purification as per standard procedures. Briefly, an overnight culture of pET23b-capsid was inoculated into fresh Luria-Bertani (LB) media containing ampicillin and chloramphenicol. At an O.D_600_ of 0.4–0.5, protein expression was induced using isopropyl-beta-D-thiogalactoside (IPTG) to a final concentration of 1 mM for 4 h at 37°C. Cells were harvested by centrifugation at 4,000 × *g* for 20 min. Cell pellet was resuspended in cold lysis buffer (50 mM NaH_2_PO_4_, 150 mM NaCl, and phenylmethanesulfonylfluoride (PMSF), pH 8.0) followed by sonication. All steps were carried out at 4°C. The crude extract was centrifuged at 17,000 × *g*. Triton-X-100 and imidazole was added to a final concentration of 1% and 20 mM respectively. Lysate was incubated with pre-washed Ni-NTA beads (Qiagen) for 2 h. The beads were washed three times with wash buffer (50 mM NaH_2_PO_4_, 150 mM NaCl, 1% Triton-X-100, 50 mM imidazole, pH 8.0). Capsid protein was eluted using 100, 250, and 500 mM imidazole buffer pH 8.0. The purified capsid protein was dialyzed overnight using a dialysis cassette with a cut-off value of 2 kDa (ThermoFisher Scientific) in dialysis buffer (50 mM NaH_2_PO_4_, 150 mM NaCl, 20 mM imidazole and 0.1% triton).

### Interaction studies

Huh-7 (human hepatocyte) cells from a confluent T150 flask were used for each interaction experiment. The cells were lysed in cold lysis buffer (50 mM NaH_2_PO_4_, 150 mM NaCl, 0.1% Triton-X-100, protease inhibitor cocktail (PIC), sodium orthovanadate and PMSF). The lysate was centrifuged at 17,000 × *g* at 4°C and supernatant was collected. 5 mg of the Huh-7 lysate protein was incubated with pre-washed Ni-NTA beads for 1 h as a pre-clearing step. 2 mg of dialyzed His-capsid protein was added to the pre-cleared Huh-7 lysate and incubated at 4°C for 2 h with continuous mixing. Prewashed Ni-NTA slurry was added to the lysate mixture and further incubated for 1 h at 4°C. The beads were washed with buffer containing (50 mM NaH_2_PO_4_, 150 mM NaCl, 0.1% Triton-X-100, 50 mM imidazole) and protein was eluted using 100 and 250 mM imidazole elution buffers. The eluates were precipitated overnight at 4°C using 10% trichloroacetic acid (TCA). Protein pellet was washed with 2% sodium acetate in ethanol, air-dried, and resuspended in 8 M urea buffer (UB).

### Filter-aided sample processing (FASP) for mass spectrometry

The TCA precipitated protein sample was resuspended in 200 μl of 8 M UB, loaded in a 3 kDa filter unit (Amicon-Millipore) and centrifuged at 14,000 × *g* for 15 min. Sample was washed by adding 200 μl of UB and centrifuged at 14,000 × *g* for 15 min. 100 μl of 0.05 M iodoacetamide (IAA) prepared in UB was added and mixed at 600 rpm for 1 min and incubated without mixing for 20 min. This was followed by centrifugation at 14,000 × *g* for 10 min. Washing was done twice by adding 100 μl of UB and centrifugation at 14,000 × *g* for 15 min. This was followed by two washes with 100 μl of 0.05 M ammonium bicarbonate (ABC) and centrifuged at 14,000 × *g* for 10 min. 40 μl ABC with trypsin (Promega V511A) (enzyme to protein ratio 1:100) was added and mixed at 600 rpm for 1 min, and then incubated in a water bath at 37°C for 16–18 h. The digested peptides were eluted at 14,000 × *g* for 10 min. Any remaining peptides were eluted with another 20–30 μl of ABC. The eluted sample was acidified with 0.1% formic acid and concentrated to 10 μl by speed vac. LC MS/MS was done using AB SCIEX Triple TOF 5600. The peptides were identified by searching the MS data against the MASCOT and PARAGON search engines. Proteins were selected for further study based on a 5% false-discovery rate cut-off and a minimum of 2-peptide-per-protein.

### Cloning of DDX3X plasmid constructs

HA-DDX3X was a gift from Robin Reed (Addgene plasmid # 44975). All plasmid constructs of DDX3X were generated in pSELECT-CHA-zeo and pSELECT-cGFP-Blas with Age-I & Bam-HI sites using the following primers: DDX3X-full length (FL) was generated using 5′-TAACCGGTATGAGTCATGTGGCAGTG-3′ (forward) and 5′-ATGGATCC GTTACCCCACCAGTCAA-3′ (reverse). DDX3X-Δ351-661 was generated using primers 5′-TAACCGGTATGAGTCATGTGGCAGTG-3′ (forward) and 5′-GGATCC ATCAGCTTCATCTAA-3′ (reverse). DDX3X-Δ385-661 was generated using 5′-TAACCGGT ATGAGTCATGTGGCAGTG-3′ (forward) and 5′GGATCCAGTAGCACTAAACATCAT 3′ reverse). DDX3X-Δ1-222Δ351-661 was generated using 5′-TAACCGGTATGTGTGCCCAAACAGGG-3′ (forward) 5′-GGATCCATATCCAACATCCGATC-3′ (reverse). DDX3X-Δ1-381 was generated using 5′-TAACCGGTATGGCTACTTTTCCTAAG-3′ (forward) 5′ATGGATCCGTTACCCCACCAGTCAA 3′ (reverse). Plasmids were constructed by standard PCR methods using Expand Hi fidelity^plus^ PCR system (Roche). Clones were verified by sequencing.

### Immunoprecipitation (IP)

For transient transfection experiments, His-tagged capsid and HA-DDX3X plasmid constructs were co-transfected in HEK293T cells using Lipofectamine-2000 (Invitrogen). 24 h post-transfection, cell lysates were prepared in cold lysis buffer (0.5% Triton-X-100 in PBS containing PIC and PMSF). The lysates were pre-cleared using protein A sepharose beads (GE healthcare) and rabbit IgG for 1 h at 4°C on a tube rotator. The supernatant was collected and incubated with capsid antibody overnight on a rotator at 4°C. Next day, pre-washed protein A sepharose beads were added and incubated for 2 h. The beads were washed with cold lysis buffer three times, and once with cold PBS. The beads were boiled in Laemmli buffer at 95°C for 5 min, loaded on SDS PAGE and transferred to PVDF membrane. Blots were probed with capsid and HA antibody. IgG light chain-specific secondary-HRP antibody was used to probe the blots. For DENV infection experiments, Huh-7 cells were plated in 3 cm dishes and infected with DENV at 5 MOI. At 24 h pi cells were collected in cold lysis buffer (0.5% Triton-X-100 in 1X TBS containing PIC, PMSF and sodium orthovanadate). The lysates were processed as described above and immunoprecipitation was performed using Capsid or DDX3X antibody. For RNAse treatment of lysates, infected Huh-7 cell lysates were treated with 200 μg/ml of bovine pancreatic RNAse A for 2 h at 4°C as per previous reports (Höck et al., 2007). The untreated and treated lysates were then used for pre-clearing with protein A sepharose beads followed by immunoprecipitation with DDX3X antibody as described above.

### Pull-down of HA-DDX3X using His-capsid

HA-DDX3X deletion constructs were transfected into HEK293T cells. 24 h p.t, cell lysates were prepared in cold lysis buffer (0.5% Triton-X-100, 20 mM Tris-Cl and 150 mM NaCl, PIC, PMSF, and sodium orthovanadate). Purified capsid protein (20 μg) was added to the 80 μg of lysate (equivalent amount of all HA-tagged protein normalized to expression levels) and incubated for 2 h at 4°C. Then 20 mM imidazole and pre-washed Ni-NTA slurry were added and incubated for 1 h at 4°C with constant mixing. The beads were washed with buffer containing 20 mM Tris-Cl,150 mM NaCl and 20 mM imidazole and boiled in 1X Laemmli buffer and processed for western blot analysis with anti-HA and capsid antibodies.

### Western blotting, siRNA transfection, real time PCR, and immunofluorescence

Cell lysates were prepared and processed for western blot analysis as described previously (Agrawal et al., 2013). siRNA targeting the human DDX3X were purchased from Dharmacon (Cat. No. L-006874-02, J-006874-07, and J-006874-18) and transfections were performed as described previously (Kumar et al., 2016).

Total RNA isolation and real-time PCR conditions for DENV viral RNA levels has been described before (Agrawal et al., 2013; Kumar et al., 2016). Briefly, total RNA was isolated using Trizol reagent (Life Technologies). cDNA was synthesized using gDNA eraser Takara cDNA synthesis kit. qRT-PCR was performed by one-step or two-step methods using either Taqman or SYBR green chemistry. Two hundred nanogram of total RNA was used to determine viral genome copy numbers using Taqman one-step qRT-PCR using primers and probe for DEN-UTR and normalized to GAPDH. DDX3X knock-down levels were verified by SYBR green chemistry using the following primers: Forward 5′ GGAGGAAGTACAGCCAGCAAAG 3′ and reverse 5′ CTGCCAATGCCATCGTAATCACTC 3′. Immunofluorescence was performed by fixing cells with methanol as described previously (Haridas et al., 2013) and stained with DDX3X, dengue envelope protein and HA antibody. Interferon beta mRNA was using the following forward and reverse primers: 5′-AAACTCATGAGCAGTCTGCA-3′ and 5′-AGGAGATCTTCAGTTTCGGAGG-3′ (Jaworska et al., 2007).

### Cell proliferation assay

Cell proliferation was assessed using Promega's CellTiter 96® AQueous One Solution cell proliferation assay kit as per the manufacturer's protocol. Briefly, knockdown of DDX3X was performed in Huh-7 cells using smart-pool siRNAs (si-DDX3X) and single siRNA (si-DDX3X-1) in 96 well plate. At 48 h p.t cells, 20 μl of the AQueous One Solution was added to each well containing 100 μl of media and incubated for 3 h. The reaction was stopped by adding 25 μl of 10% SDS. The supernatant was diluted 1:5 in PBS and absorbance was measured at 490 nm. OD_490_ from media alone with the AQueous One Solution was used to subtract background.

### Flow cytometry

Cells were collected by trypsinization and washed with FACS buffer (PBS+0.25% FBS). The fixable viability stain (BD EF-780) was added at 1:1,000 dilution and cells were incubated at RT for 10 min followed by washing with FACS buffer. Cells were centrifuged at 700 × *g* for 5 min and fixed in 3% PFA for 10 min on ice. Subsequently all steps were carried out at 4°C. Cells were washed once with FACS buffer and permeabilized with IMF buffer (20 mM HEPES pH 7.5, 0.1% triton-X 100, 150 mM NaCl, 5 mM EDTA, 0.02% sodium azide) for 20 min. The cells were washed with FACS buffer and pelleted by centrifugation. Primary anti-dengue envelope antibody conjugated to APC was prepared in IMF buffer at a dilution of 1:400, added to the cells and incubated for 1 h. The cells were washed twice with FACS buffer and resuspended in 1X PBS and acquired in BD FACS Canto-II. 50,000 cells were acquired in forward vs. side scatter in a linear scale. Data was analyzed using FlowJo (Treestar).

### Statistical analysis

GraphPad Prism software was used for all graphical representations and statistical analysis. All experiments were performed with two or three technical triplicates and all experiments have been performed two or more times. Non parametric, two tailed *t*-tests were performed to calculate *p*-values. ^*^p < 0.05, ^**^p < 0.005, and ^***^p < 0.0005.

## Results

### Identification of DDX3X as a DENV capsid-interacting protein

Previous studies have identified a number of cellular proteins that interact with dengue capsid using yeast-two-hybrid system and co-immunoprecipitation studies (Limjindaporn et al., 2007; Netsawang et al., 2010; Balinsky et al., 2013; Mairiang et al., 2013). Interestingly, these studies focused on the nuclear proteins that bound to capsid and elucidated the mechanism of capsid transport to the nucleus and possible link of host gene regulation due to the interaction of host proteins with capsid (Bhuvanakantham et al., 2009; Balinsky et al., 2013). To identify cytosolic proteins that interact with DENV capsid, we expressed and purified DENV capsid protein without the membrane anchor domain in a bacterial expression system (Figure S1). The purified His-tagged capsid protein was allowed to interact in-solution with cell lysates prepared from Huh-7 cells. Ni-NTA beads were added to the reaction mix to pull down the capsid and any other interacting proteins. As capsid protein expressed in bacteria lacks post-translational modifications compared to the capsid protein in mammalian cells, it is likely that many interacting partners whose interaction depends on post-translational modifications would be missed by our approach. Nevertheless, the proteins bound to the beads were eluted and were digested with trypsin and processed for LC MS/MS to identify interacting proteins. The identification of peptides was performed by searching the Mascot and Paragon search engines and proteins were selected with a 5% false-discovery rate cut-off and a minimum of 2-peptide-per-protein. Using these criteria, 16 proteins were identified as potential DENV capsid interacting proteins (Table 1). Of these, DDX3X, which was identified consistently in four experiments, with the highest number of peptides was selected for further characterization.

**Table 1:** List of identified potential capsid interacting proteins from mass spectrometry analysis.

**Identified protein**	**No. of peptides (95% confidence)**
DDX3X: DEAD (Asp-Glu-Ala-Asp) box, X-Linked, ATP dependent RNA helicase 3	25
Heterogeneous nuclear ribonucleoprotein Q	13
cDNA FLJ54373, highly similar to 60 kDa heat shock protein, mitochondrial	9
60S ribosomal protein L35	7
Heterogeneous nuclear ribonucleoprotein A0	6
Heterogeneous nuclear ribonucleoprotein A1	6
Histone H1.4	6
Heterogeneous nuclear ribonucleoprotein U	4
40S ribosomal protein S13	3
40S ribosomal protein S11	3
40S ribosomal protein S15a	2
40S ribosomal protein S8	2
60S ribosomal protein L23a	2
Pyrroline-5-carboxylate reductase 2	2
cDNA, FLJ94136, highly similar to Homo sapiens synaptotagmin binding, cytoplasmic RNA interacting protein (SYNCRIP)	2
cDNA FLJ53662, highly similar to Actin, alpha skeletal muscle	2

### Domain mapping of DENV capsid and DDX3X interaction

**Figure 1:**
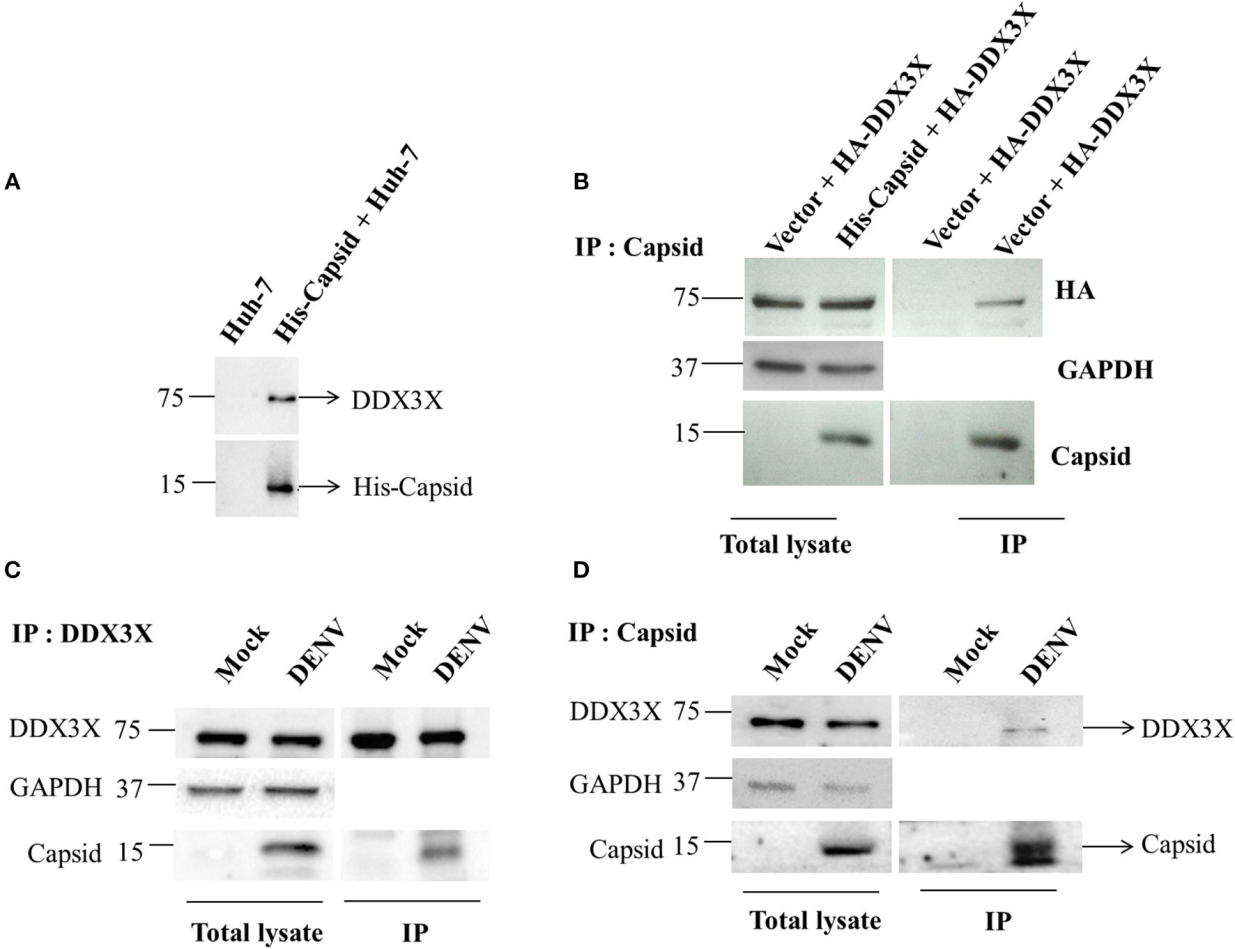
Capsid coimmunoprecipitates with DDX3X from cell lysates. **(A)** In-solution interaction of purified His-tagged DENV capsid and Huh-7 lysate was followed by pull-down of interacting proteins using Ni-NTA beads. Pull down with Huh-7 lysate alone is used as a control. **(B)** His-tagged DENV capsid and HA-DDX3X were co-transfected in HEK293T cells. 24 h p.t. lysate was prepared and capsid was immunoprecipitated using DENV capsid antibody. Total lysate and immunoprecipitated proteins were analyzed by western blots probed for capsid, HA and GAPDH. **(C,D)** Huh-7 cells were infected with DENV at an MOI of 5. At 24 h pi mock and DENV-infected Huh-7 cell lysates were prepared, endogenous DDX3X **(C)** or DENV capsid **(D)** was immunoprecipitated from total lysates using DDX3X or DENV capsid antibody respectively. Total lysate and immunoprecipitated proteins were analyzed by western blots probed for capsid and DDX3X.

**Figure 2:**
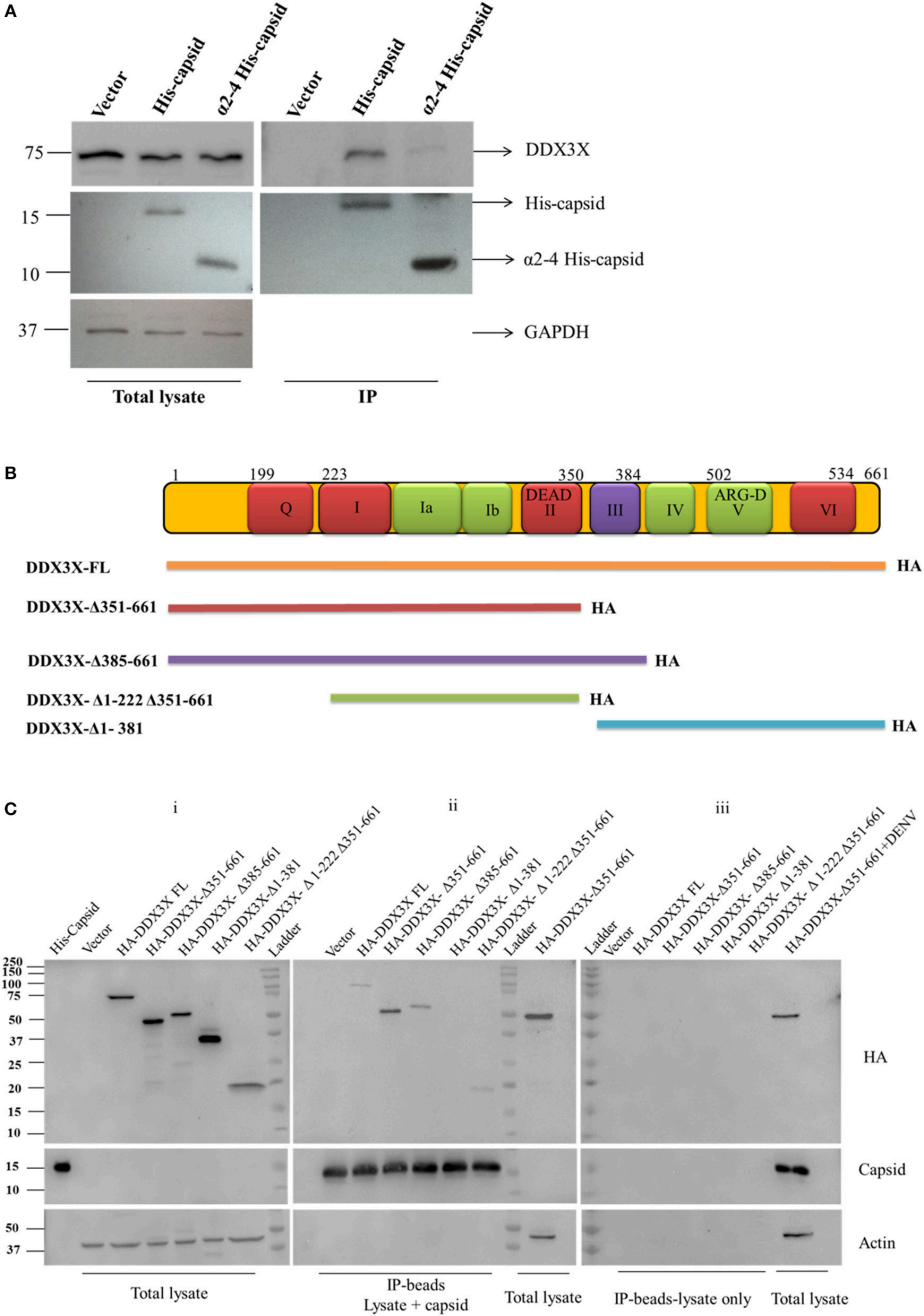
Co-immunoprecipitation and pull-down of recombinant capsid and DDX3X. **(A)** Full length His-capsid and α2-4 His-capsid were transfected in HEK293T cells and lysates were prepared 24 h p.t. Lysates were used for IP with capsid antibody and blots were probed for capsid, DDX3X and GAPDH. **(B)** Map of the HA-DDX3X deletion constructs. **(C)** Total lysates were prepared from HEK293T cells transfected with HA-DDX3X-FL and HA-DDX3X deletion constructs at 24 h p.t. Purified His-capsid was added to the lysate for in-solution interaction and protein complexes were pulled down by Ni-NTA beads. Lysates incubated with Ni-NTA beads only were used as negative control. The beads were washed, boiled in Laemmli buffer and loaded on SDS PAGE and probed for HA-tag, capsid and Actin. In the first lane of blot (i) purified His-capsid was loaded as a positive control for capsid antibody. Total lysate of HEK293T cells transfected with HA-DDX3X-Δ351-661 and total lysate of HEK293T cells transfected with HA-DDX3X-Δ351-661 and infected with DENV was loaded as positive controls respectively in blots (ii) and (iii) for HA, capsid and actin antibodies.

We validated the mass spectrometry data by confirming the pull-down of DDX3X in the eluates from capsid and Huh-7 lysate interaction experiment by western blot analysis and identified DDX3X in interactions that had both the capsid protein and cell lysates but not with beads that were incubated with Huh-7 cell lysate without capsid (Figure 1A). We next used HEK293T cells for co-transfection of His-capsid and HA-DDX3X plasmids. Capsid-DDX3X interaction was verified by performing immunoprecipitation (IP) of capsid and western blot analysis for DDX3X. DDX3X was found to co-immunoprecipitate with capsid (Figure 1B). We next performed IP with either DDX3X antibody or capsid antibody in cell lysates prepared from Huh-7 cells infected with 5 MOI of DENV2. We observed co-IP of capsid and DDX3X respectively demonstrating the interaction of dengue capsid and DDX3X in the context of dengue infection (Figures 1C,D). The interaction of DDX3X with capsid was unaffected by pre-treatment of lysates with RNAse suggesting that the interaction was not bridged by RNA (Figure S2). Earlier studies with Hepatitis C virus core protein have shown that the N-terminal region of the core protein is sufficient for interaction with DDX3X (Owsianka and Patel, 1999). We generated truncated versions of DENV2 capsid based on the alpha-helical regions (Ma et al., 2004). However, we were able to detect expression of only the capsid construct lacking the first α-helix (α2-4-capsid) (Figure S3). Next, we transfected either full-length or α2-4-capsid constructs into HEK293T cells and verified co-IP of endogenous DDX3X with capsid antibody. As expected, DDX3X pull-down was observed with full-length capsid and the same was drastically reduced with α2-4-capsid suggesting that the N-terminal 45 amino acids of DENV capsid is required for interaction with DDX3X (Figure 2A). To further identify the capsid-interacting region in DDX3X, we generated HA-tagged DDX3X constructs with C-terminal deletions (DDX3X-Δ351-661, DDX3X-Δ385-661), N-terminal deletion (DDX3X-Δ1-381) and a double deletion mutant (DDX3X-Δ1-222, Δ351-661) and the full-length DDX3X (DDX3X-FL) (Figure 2B). We verified the expression of these deletion constructs in HEK293T cells and observed that the expression of DDX3X-Δ1-381 was highest, followed by DDX3X-Δ351-661. DDX3X-FL and DDX3X-Δ385-661 constructs had comparable expression levels. The double deletion mutant (DDX3X-Δ1-222, Δ351-661) had the lowest expression compared to all other constructs (Figure 2C, i). As we faced technical difficulties with co-immunoprecipitation of capsid using HA antibodies from infected cells, these HA-tagged constructs were expressed in HEK293T cells and equal quantity of lysates (normalized to HA-DDX3X expression levels) containing the recombinant HA-tagged proteins were incubated with purified His-capsid by in-solution interaction method as described in methods section. His-capsid was pulled down using Ni-NTA and co-elution of capsid protein was observed with all the HA-DDX3X constructs except the N-terminal deletion mutant (DDX3X-Δ1-381) (Figure 2C, ii). It is important to note that pull-down of HA-tagged DDX3X was observed in the presence of endogenous DDX3X present in the lysate which would act as a competitor for binding to capsid protein. Therefore, the low amounts of HA-DDX3X observed cannot be interpreted as weak interaction. The same lysates incubated with Ni-NTA beads alone failed to pull down any of the HA-tagged proteins ruling out non-specific interaction with the beads alone (Figure 2C, iii). The double deletion mutant (DDX3X-Δ1-222, Δ351-661) was capable of co-eluting with capsid, albeit at a lower levels relative to other constructs, and the DDX3X-Δ351-661 was most efficient in interaction with capsid under these conditions. Therefore, our data indicate that the N-terminal domain consisting of amino acids 223-350 is the minimal domain required for efficient interaction of DDX3X with DENV capsid.

### DENV down-regulates DDX3X at later stages of infection

**Figure 3:**
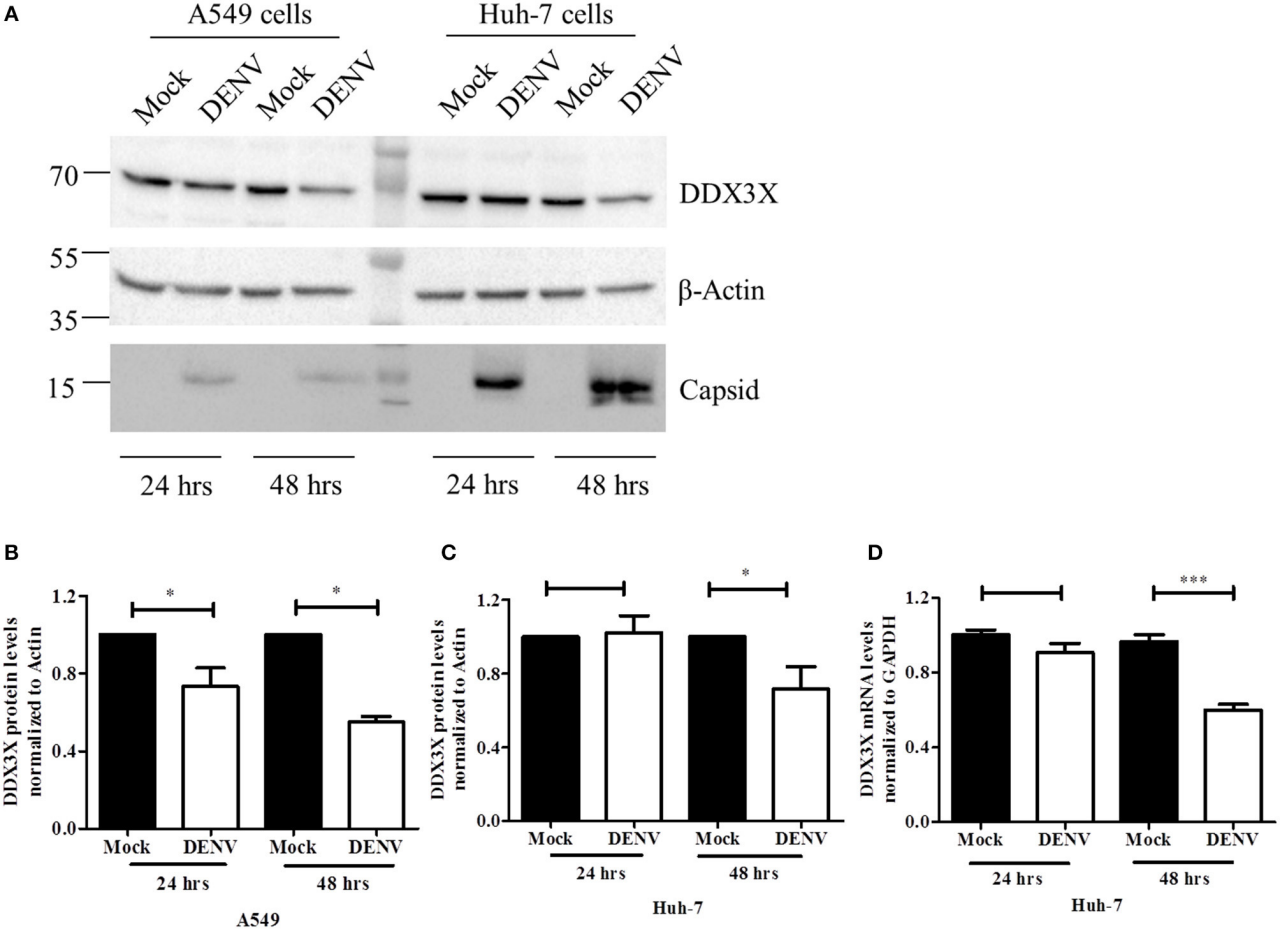
DENV down-regulates DDX3X at later stages of infection. A549 and Huh-7 cells were infected with DENV at 3 MOI. Cells were collected at 24 and 48 h pi for either lysate preparation or for total RNA isolation. **(A)** Cell lysates were used for western blot analysis for DDX3X, GAPDH and capsid in both cell lines. **(B)**. Densitometry quantitation of the blots for A549 cells (*n* = 4). **(C)** Quantitation of blots for Huh-7 cells (*n* = 4). **(D)** Total RNA was used to estimate mRNA levels of DDX3X by real-time PCR from 3 independent experiments performed in triplicates (*n* = 9). Data is represented as mean ±SEM. ^*^P < 0.05 and ^***^P < 0.0005, ns = not significant.

The role of DDX3X in viral infections is poorly characterized. DDX3X has been shown to act as either a necessary factor or an inhibitor in viral replication (Yedavalli et al., 2004; Chang et al., 2006; Kalverda et al., 2009; Angus et al., 2010; Oshiumi et al., 2010a; Garbelli et al., 2011a; Chahar et al., 2013; Lai et al., 2013; Li et al., 2014, 2015; Pène et al., 2015). We analyzed the effect of DENV infection on DDX3X protein levels in different cell lines. A549 and Huh-7 cells were infected with DENV and DDX3X levels were monitored by western blot analysis at 24 and 48 h pi. We observed a significant decrease in DDX3X protein levels at 48 h pi in both the cell lines (Figures 3A–C). We further verified if the reduction in the DDX3X protein levels is reflected at the mRNA levels by measuring the transcripts of DDX3X in dengue infection in Huh-7 cells. We observed a significant reduction in the mRNA levels of DDX3X at 48 h pi suggesting that DENV infection suppresses the expression of DDX3X at mRNA level at later stages of infection (Figure 3D). The reduction in DDX3X levels were further confirmed by immunofluorescence analysis where DDX3X levels were lower in infected cells at 48 h pi however there was no significant change in distribution of DDX3X in infected cells and we observed no co-localization with viral envelope (Figure S4). Colocalization studies with viral capsid could not be performed as both DDX3X and capsid antibodies were raised in rabbit.

### DDX3X inhibits DENV replication

**Figure 4:**
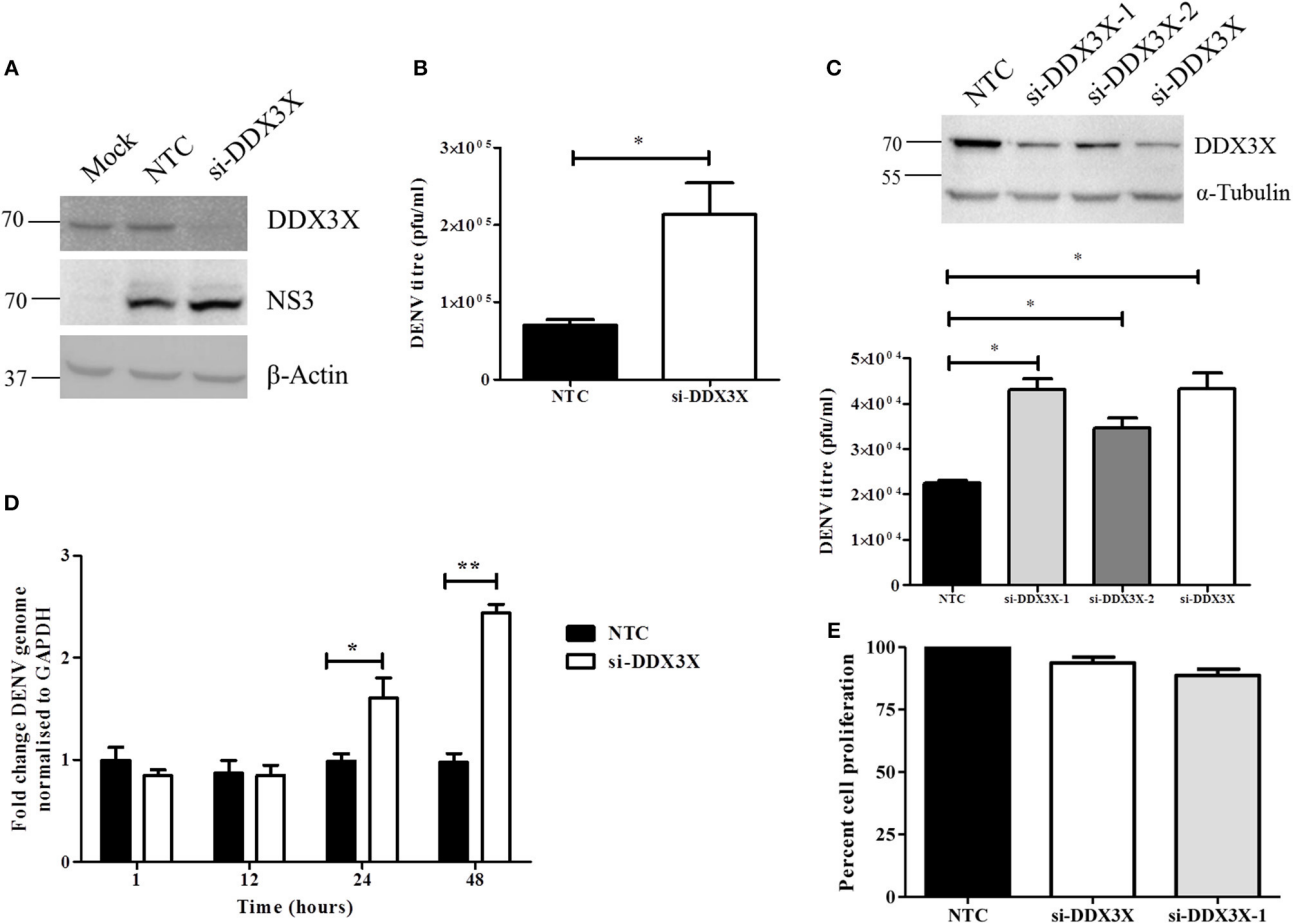
Knockdown of DDX3X enhances DENV infection. Knockdown of DDX3X was performed in Huh-7 cells using smartpool siRNAs (si-DDX3X) and at 48 h p.t cells were infected with DENV at 1 MOI. Viral titres and RNA levels were verified at 24 h p.i. **(A)** Western blot analysis to verify knockdown of DDX3X. β-actin level is shown as a loading control and DENV-NS3 antibody was used to detect infection levels. **(B)** Viral titers were estimated by plaque assay. **(C)** DDX3X expression was knocked down by a single siRNA (si-DDX3X-1 or si-DDX3X-2) or using smartpool siRNAs (si-DDX3X) in Huh-7 cells and 48 h p.t. cells were infected with DENV at 1 MOI. Upper panel shows the western blot for knockdown efficiency. Lower panel shows viral titres estimated at 24 h pi. Figure is representative of three independent experiments performed with triplicate samples **(D)** Kinetics of infection in Huh-7 cells after DDX3X knockdown. Cells were collected at 1, 12, 24, and 48 h pi and DENV genome levels were estimated by real-time PCR. Data from two independent experiments performed with triplicate samples (*n* = 6) is represented as mean ± SEM. **(E)** Knockdown of DDX3X was performed in Huh-7 cells using smartpool siRNAs (si-DDX3X) and single siRNA (si-DDX3X-1) and cell proliferation was assessed as described in the methods section at 48 h post-transfection. ^*^P < 0.05 and ^**^P < 0.005.

To further assess the role of DDX3X in DENV infection, we performed the knock-down of DDX3X expression using siRNAs in Huh-7 cells. Cells were treated with either DDX3X smart-pool siRNAs or a non-targeting control (NTC) siRNA followed by infection with DENV2. Supernatants were collected at 24 h pi for plaque assay and total RNA was isolated from cells for RT-PCR. Knockdown of DDX3X was confirmed by western blot analysis with cell lysates (Figure 4A). There was about three-fold increase in viral titers in the supernatants of cells where DDX3X expression was knocked down as compared to the NTC (Figure 4B). We further confirmed this observation by using single siRNAs (siDDX3X-1 and siDDX3X-2) to knock-down DDX3X in order to rule out off-target effects of smart-pool siRNAs (siDDX3X). A similar increase in viral titers was observed when individual siRNAs were used to knock-down DDX3X expression (Figure 4C) further confirming that the increase in viral titers is not due to the off-target effect of smart pool siRNAs. The increase in viral titers due to DDX3X knock-down suggested enhanced viral entry, replication or egress due to reduced DDX3X expression. To further identify the stage of DENV life cycle that DDX3X may be involved in, we performed time-course experiments and evaluated the DENV genome levels at 1, 12, 24, and 48 h pi in Huh-7 cells transfected with NTC or DDX3X siRNAs. We observed around 1.4 fold increase in DENV RNA levels at 24 h pi which further increased to 2.5-fold at 48 h pi in DDX3X knock-down cells as compared to NTC (Figure 4D). The viral RNA levels at 1 h pi did not show any difference between si-DDX3X and NTC samples indicating that the effect observed is not due to enhanced viral entry. The knock-down of DDX3X by siRNAs did not affect cell proliferation (Figure 4E). Similar results were obtained with A549 cells where knock-down of DDX3X led to enhanced DENV RNA levels and viral titers as compared to NTC (Figures S5A–D). These results clearly suggest that DDX3X may exert its antiviral effect at a post-entry stage which may have a direct or indirect effect on DENV RNA replication.

**Figure 5:**
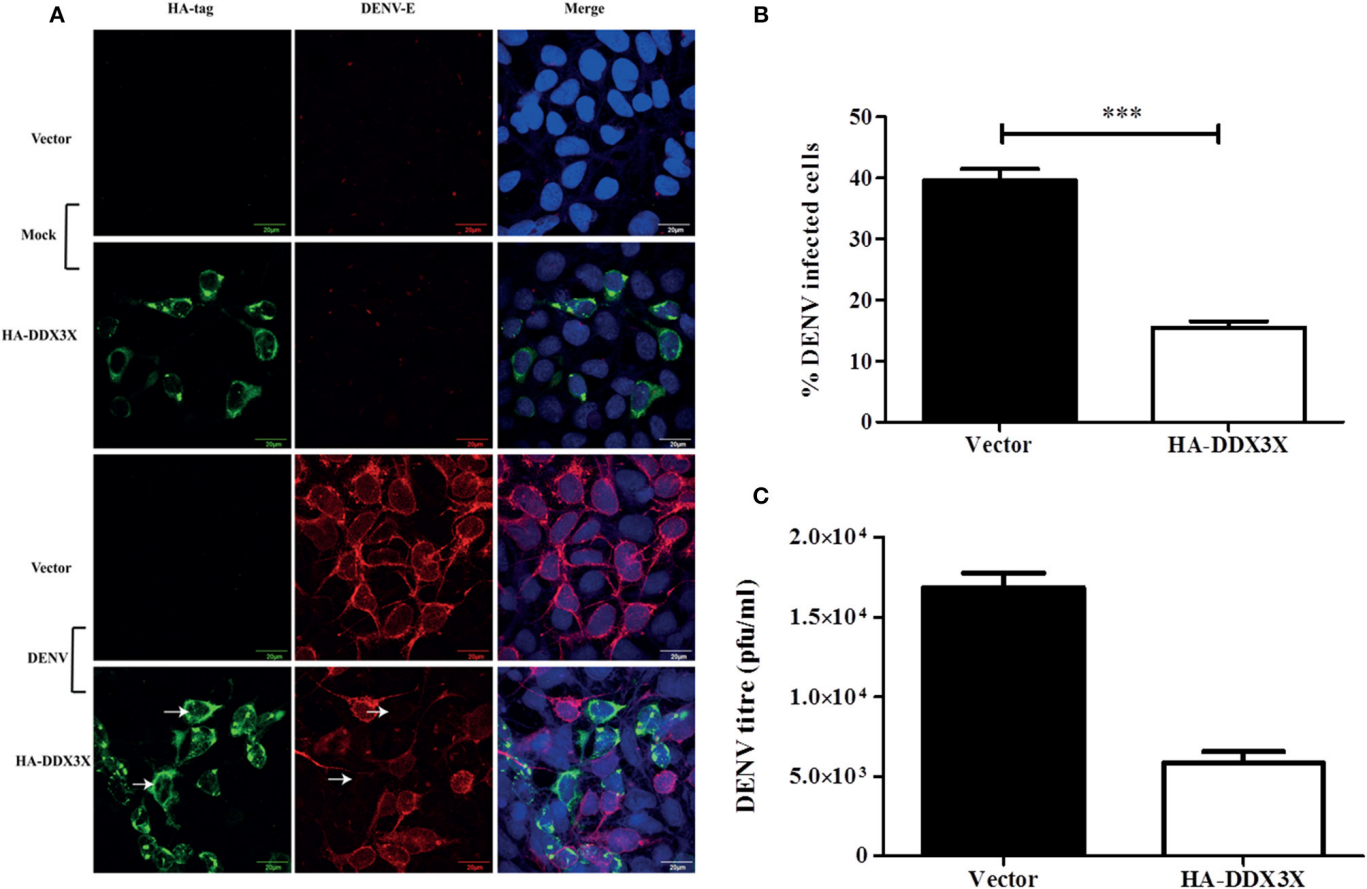
DDX3X overexpression suppresses dengue infection. **(A)** HA-DDX3X plasmid or the vector was transfected in HEK293T cells. 24 h p.t. cells were infected with DENV at 5 MOI. Cells were fixed and stained for dengue envelope and HA at 24 h pi. **(B)** Percentage of infected cells was counted in ten fields each in three independent experiments (~3000 cells were counted) **(C)** Viral titres were estimated by plaque assay at 24 h pi. Data is represented as mean ± SEM. ^***^P < 0.0005.

As silencing DDX3X expression led to increased DENV titers, we speculated that overexpression of DDX3X would lead to an enhanced antiviral state and inhibit DENV infection. We tested this by transfecting HA-DDX3X plasmid and its vector in HEK293T cells. At 24 h p.t, cells were fixed and stained with HA and DENV envelope antibody. Viral titers in the supernatants were measured by plaque assay. DENV infection was absent in cells expressing HA-DDX3X while un-transfected cells showed DENV positivity (Figure 5A). HA-DDX3X overexpression led to a 50% reduction in DENV infection (Figure 5B), which further correlated with a five-fold reduction in viral titers (Figure 5C). Similar results were obtained in Huh-7 cells transfected with HA-DDX3X where we observed 50% reduction in both viral titers and DENV RNA levels under DDX3X overexpression conditions (Figures S6A,B). Our results with DDX3X knock-down and overexpression suggest an antiviral role for DDX3X in DENV infection.

### The N-terminal 1-350 aa domain of DDX3X is required for the antiviral activity

**Figure 6:**
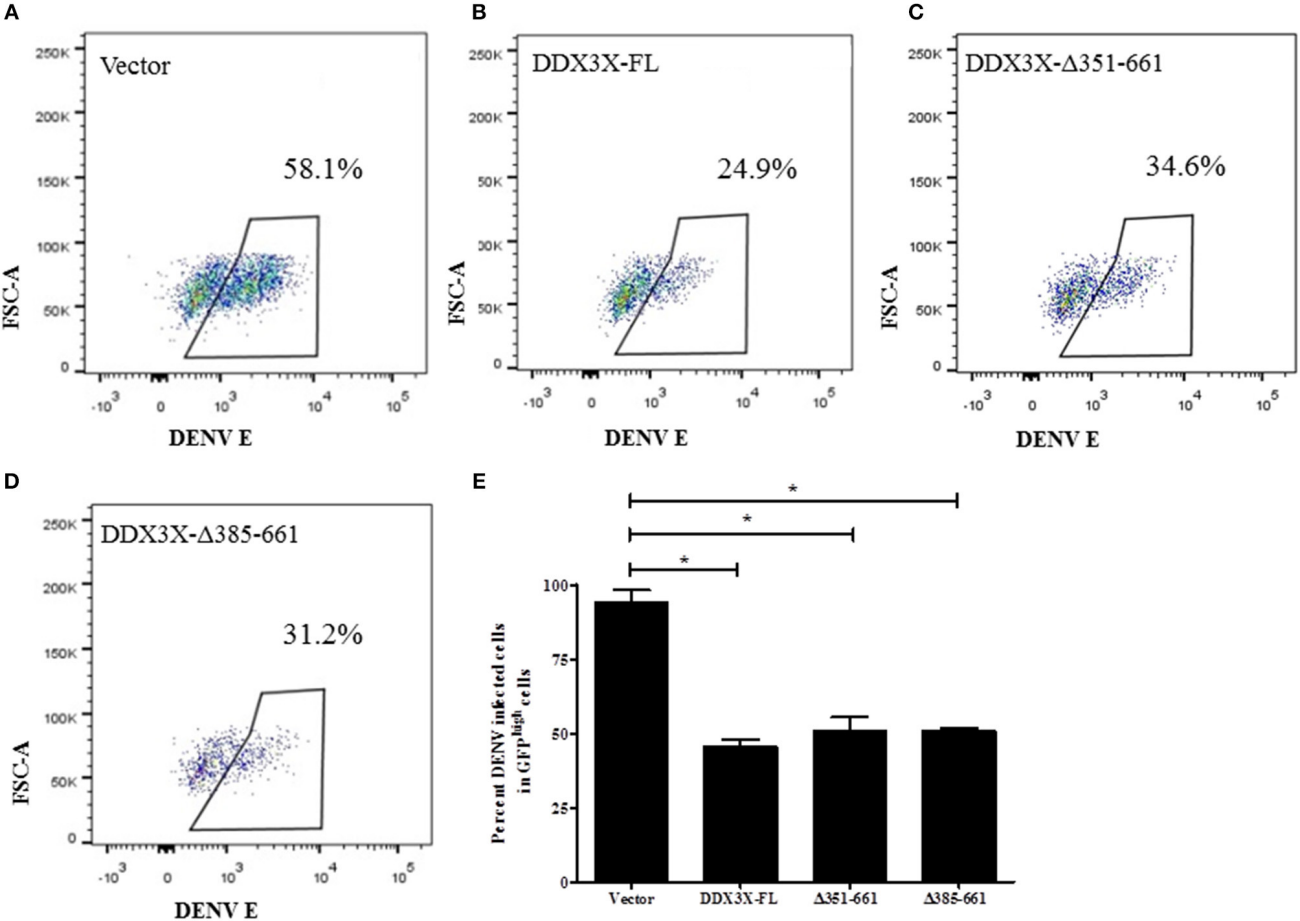
Domain mapping of DDX3X antiviral activity. GFP tagged-DDX3X plasmids were transfected in HEK-293T cells and at 24 h p.t, cells were infected with DENV at 5 MOI. Cells were fixed and stained at 24 h pi with dengue envelope antibody. Cells were acquired in FACS and GFP signal was used to identify transfected cells. **(A–D)** Representative plots for percent DENV positive in GFP^high^ cells for each GFP-DDX3X construct. **(E)** Quantitation of DENV positivity in GFP^high^ for all GFP-DDX3X constructs. The experiment was performed twice with two replicates in each. Data is represented as mean ±SEM. ^*^P < 0.05.

DDX3X performs multiple functions due to its multiple functional domains, which include the RNA helicase, ATPase and RNA binding activities (Garbelli et al., 2011b; Soto-Rifo and Ohlmann, 2013; Valiente-Echeverría et al., 2015). To further verify the domain required for the antiviral activity of DDX3X by flow cytometry we created truncations in the DDX3X protein by generating the following GFP-tagged constructs: full length DDX3X (DDX3X-FL) and constructs lacking the c-terminal domains (DDX3X-Δ351-661, DDX3X-Δ385-661). The plasmids were transfected in HEK293T and at 24 h post-transfection, the cells were infected with DENV at 5 MOI and at 24 h pi cells were fixed and DENV infection in GFP^high^ cells was assessed by staining with DENV-E antibody conjugated with allophycocyanin (APC) using flow cytometry (Figure S7). Cells expressing GFP-tagged-DDX3X-FL showed a 50% decrease in infection as compared to the GFP-vector alone (Figures 6A,B). The antiviral activity of GFP-DDX3X was less as compared to HA-DDX3X (Figure 4) most probably due to differences in the expression levels of these constructs and also due to altered sub-cellular localization of GFP-DDX3X most likely due to the larger GFP-tag (Figure S8). Cells expressing DDX3X-Δ351-661 (34.6%) and DDX3X-Δ385-661 (31.2%) also showed a significant inhibition of DENV infection (Figures 6C,D), although the effect was slightly less than full-length DDX3X (Figure 6E). Overexpression of any of these constructs had no effect on cell viability (Figures S9A–D). We also generated the N-terminal deletion construct GFP-DDX3X-Δ1-381 which was found to localize to the nucleus suggesting that the c-terminal region of the protein is necessary for proper localization of the protein. Therefore, although this construct was useful in *in vitro* assays to demonstrate binding to capsid, the same was not appropriate for performing cellular interactions and antiviral functions due to altered localization. As the loss of aa 351-661 retained the antiviral activity, these results indicate that the N-terminal region of DDX3X, 1-350 aa, which contains the motifs Q, I, Ia, Ib, and DEAD box is sufficient for the antiviral functions of DDX3X. Such an effect may be expected as this region contains conserved motifs that are involved in ATP and RNA binding (Bol et al., 2015).

In a previous report, the antiviral function of DDX3X was attributed to its role in the induction of interferon-β as knock-down of DDX3X led to suppression of IFN-β production and increase in DENV titers (Li et al., 2015). To confirm this under our infection conditions, we measured the mRNA levels of IFN-β in cells with si-DDX3X transfection. We did not observe any significant difference in the basal levels of IFN-β transcript when DDX3X expression was suppressed in Huh-7 cells. Moreover, induction of IFN-β was not impaired upon dengue infection in both NTC and si-DDX3X cells indicating that DDX3X was not essential for induction of IFN-β in DENV infection or the residual levels of DDX3X present in knock-down cells may be sufficient for IFN-β production (Figure S10). These results indicate that the antiviral effect of DDX3X in dengue infection is independent of interferon induction pathways.

## Discussion

Previous studies have shown the role of DDX3X in viral infections and in innate immune signaling (Yedavalli et al., 2004; Chang et al., 2006; Kalverda et al., 2009; Angus et al., 2010; Oshiumi et al., 2010a; Garbelli et al., 2011a; Chahar et al., 2013; Lai et al., 2013; Li et al., 2014, 2015; Pène et al., 2015). We have identified DDX3X as a DENV capsid-interacting protein and further confirm its anti-viral role in DENV infection. We show that the N-terminal 45 amino acids including the α-helix-1 of DENV-C was essential for interaction with DDX3X. Interestingly, HCV core protein was also shown to interact with DDX3X via its N-terminal domain and abrogation of DDX3X and HCV core interaction by mutagenesis had no effect on viral replication and infectious virus production indicating that HCV core and DDX3X interaction may have an indirect effect on host functions (Angus et al., 2010). In addition, DDX3X was also identified as a host factor in HCV infection by a genome-wide genetic screening and was shown to be involved at late stages of viral replication (Li et al., 2009). DDX3X was shown to sequester HCV RNA to lipid droplets and this was proposed to be an immune evasion strategy (Ariumi et al., 2011). Although HCV is a member of the family *Flaviviridae*, it belongs to a different genus (Hepacivirus) and has a different mode of RNA replication. The HCV core was shown to bind c-terminal domain of DDX3X and knock-down of DDX3X led to reduction in viral replication which is contrary to our observations with dengue virus. Therefore, these viruses may utilize DDX3X in different ways. The role of DDX3X in HIV replication is one of the well-characterized examples. DDX3X along with its binding partner Chromosome Maintenance-1 (CRM-1) was involved in the nuclear export of HIV RNA by Rev-Rev Response Element (RRE) complex (Yedavalli et al., 2004). In addition, DDX3X was also shown to promote translation initiation of HIV mRNA (Soto-Rifo et al., 2013). DDX3X been shown to be required for viral replication in the case of murine norovirus (Yedavalli et al., 2004; Angus et al., 2010; Vashist et al., 2012). DDX3X has also been shown to act as an antiviral protein owing to its involvement in the upstream events leading to IFN-β induction (Schröder et al., 2008; Soulat et al., 2008; Oshiumi et al., 2010b). Therefore, in contrast to its proviral role in the case of HCV and HIV, DDX3X has been shown to play an antiviral role in vaccinia virus, hepatitis B virus and vesicular stomatitis virus infections (Oshiumi et al., 2010b; Wang and Ryu, 2010; Schroder, 2011). In the case of flaviviruses, DDX3X and other components of P bodies and stress granules were shown to be redistributed to viral replication compartments in West Nile virus infected cells although the relevance of this finding is yet to be determined (Chahar et al., 2013). In the case of Japanese encephalitis virus, DDX3X knock-down led to inhibition of viral titers, however, overexpression of DDX3X or any of the mutants under knock-down conditions suggested that the helicase activity of DDX3X is involved in JEV replication in BHK-21 cells. DDX3X was shown to play a role in translation of JEV proteins by interacting with viral non-structural proteins and 5′ and 3′ untranslated regions of viral RNA (Li et al., 2014). As JEV is a neurotropic virus, these observations need further validation in cells of central nervous system or at least in immortalized human cell lines. In a siRNA screening to identify DEAD-box family genes involved in DENV infection, Li et. al., identified DDX3X and DDX50 as the two DEAD-box family members which, when suppressed by siRNAs, led to significant upregulation of viral replication and DDX25 and DDX26 knock-down led to downregulation of DENV replication (Li et al., 2015). We have made a similar observation in our study where knock-down of DDX3X led to increase in viral titers and intracellular viral RNA levels without affecting viral entry and overexpression of DDX3X led to inhibition of virus replication. We were not successful in our attempts to purify DDX3X protein in prokaryotic expression system, therefore, a direct binding between DDX3X and dengue capsid could not be demonstrated. Nevertheless, we show by indirect methods that the motifs within the N-terminal region are important for the anti-viral role of DDX3X, since C-terminal deletions up to 350 a.a retained anti-viral functions of DDX3X. The N-terminal region of the protein consists of motifs Q, I, Ia, Ib, and DEAD box, which are involved in ATP and RNA binding (Cordin et al., 2006; Henn et al., 2012; Bol et al., 2015), and thus may be important in anti-viral response in the cell. This clearly demonstrates the antiviral function of DDX3X in cells infected with dengue virus and we show downregulation of DDX3X at later stages of DENV infection which could be an immune evasion strategy.

Huh-7 cells are deficient in TLR3-mediated pathway and rely on RIG-I pathway for induction of type I interferons (Li et al., 2005). We observed that DDX3X knock-down did not hamper the induction of IFN-β suggesting that reduction in type I interferons is not a reason for higher viral titers observed in these cells. We speculate that DDX3X may mediate anti-viral effects independent of IFN-β response by other stress response pathways. We have shown recently that drugs that induced autophagy acted as potent inhibitors of DENV replication (Medigeshi et al., 2016). Therefore, it would be interesting to investigate the role of DDX3X in autophagy. DDX3X is a phosphorylation target of TANK-binding kinase-1 (TBK-1), which has been shown to be a crucial bridge between innate immune responses and autophagy (Weidberg and Elazar, 2011; Pilli et al., 2012). It is possible that DDX3X may regulate autophagy through some of its interacting partners such as TBK-1, which warrants further investigation.

Recent reports have suggested a prominent role for DDX3X in stress granule formation and many viruses have been shown to hijack DDX3X to modulate/utilize this function (Chahar et al., 2013). We have not investigated the role of stress granules in our study. However, it should be noted that the list of proteins identified by mass spectrometry included ribonucleoproteins (RNPs) hnRNPQ, hnRNPA0, hnRNPA1 and hnRNPU of which all except hnRNPA0 have been shown to be components of stress granules and P bodies (Guil et al., 2006; Gallois-Montbrun et al., 2007; Quaresma et al., 2009). Recent reports have demonstrated the role of ribonucleoproteins in dengue virus replication as NS-1 and capsid-interacting partners (Chang et al., 2001; Brunetti et al., 2015; Dechtawewat et al., 2015; Diwaker et al., 2016). Further studies are required to understand if the hnRNPs identified in our study also play a similar role in DENV infection. It is plausible that DDX3X is part of a multifunctional protein complex involving RNPs, which interact with DENV capsid and future studies may focus on probing this further. The other capsid-interacting proteins identified in our study are various 60S and 40S ribosomal proteins which have been shown to be involved in ribosomal biogenesis. A putative protein which is highly similar to 60 kDa heat shock protein was also identified in this study. Interestingly, HSP60 has been shown to be involved in viral infections such as rotavirus, hepatitis B virus and dengue virus by either modulating apoptotic pathways or immune responses (Tanaka et al., 2004; Padwad et al., 2009; Chattopadhyay et al., 2017). In addition, as in this study, previous reports have also identified histone proteins as binding partners of DENV capsid further indicating that the list of proteins that we have identified are relevant for DENV biology (Colpitts et al., 2011). The relevance of other identified proteins such as the mitochondrial protein Pyrroline-5-carboxylate reductase 2 which catalyzes the conversion of pyrroline-5-carboxylate to proline, and other putative proteins similar to synaptotagmin binding, cytoplasmic RNA interacting protein (SYNCRIP) and skeletal muscle α-actin remains to be explored.

## Author contributions

RK performed experiments, analyzed data and wrote the manuscript. NS performed experiments and analyzed data. MA analyzed data and provided critical inputs. AP contributed reagents, designed experiments, analyzed data and provided critical inputs. GM conceived the study, designed and performed experiments, analyzed data and wrote the manuscript. All authors have reviewed the final version of the manuscript.

### Conflict of interest statement

The authors declare that the research was conducted in the absence of any commercial or financial relationships that could be construed as a potential conflict of interest.

## Supplementary material

Figure S1 Purification of His-capsid protein. **(A)** Purification of the His-capsid protein as described in methodology using Ni-NTA beads. **(B)** Western blot showing purified His-capsid probed using anti-His antibody.

Figure S2 Interaction between capsid and DDX3X is not mediated via RNA. Huh-7 cells were infected with DENV at an MOI of 5. At 24 h pi mock and DENV-infected Huh-7 cell lysates were prepared and treated with RNAse prior to immunoprecipitation. DDX3X was immunoprecipitated from total lysates (untreated) and RNAse-treated lysates using DDX3X antibody at 4°C. Total lysate and immunoprecipitated proteins were detected by western blots.

Figure S3 Deletion constructs of capsid. **(A)** Graphical representation of His-capsid deletion constructs. **(B)** HEK293T cells were transfected with plasmids encoding various capsid constructs. Total lysates were prepared at 24 h p.t. and expression of recombinant His-tagged proteins were analyzed by western blotting with capsid antibody. GAPDH levels are shown as loading controls.

Figure S4 DENV infection downregulates DDX3X in Huh-7 cells at later stages of infection. **(A)** Huh-7 cells were infected at 1 MOI and fixed at 24 and 48 h pi. Cells were stained for DDX3X (red), dengue envelope (green) and nuclei with DAPI (blue). **(B)** The fluorescence intensity of DDX3X staining was quantitated using Fluoview software. Quantitation of DDX3X fluorescence intensity (AU) at 24 and 48 h pi. (*n* = 15) ^***^*P* < 0.0005.

Figure S5 DDX3X downregulation enhances DENV infection in A549 cells. **(A)** Cells were transfected with si-NTC or si-DDX3X and at 48 h p.t. knock-down efficiency was measured by real-time PCR from total RNA. **(B)** Cells transfected with si-NTC or si-DDX3X were infected with DENV at 3 MOI at 48 h p.t. Viral titres were estimated at 24 h pi by plaque assay and viral genome levels were analyzed by real-time and normalized to GAPDH **(C)** Data represents three independent experiments performed with triplicates samples (*n* = 9). (**D)** Viral entry was assessed in DDX3X knockdown cells. Cells were collected after 1 h of virus adsorption and viral genome levels was analyzed by real-time PCR. Data presented are from two independent experiments performed with three replicates each (*n* = 6). Data is represented as mean ±SEM. ^**^*P* < 0.005 and ^***^*P* < 0.0005, ns = not significant.

Figure S6 Overexpression of HA-DDX3X in Huh-7 cells inhibits virus replication. HA-DDX3X was transfected in Huh-7 cells, and at 24 h p.t, cells were infected with DENV at 3 MOI and viral titers were measured by plaque assay at 24 h pi **(A)** DENV viral genome levels were estimated by real-time PCR. Data is from three independent experiments performed in triplicates (*n* = 9). **(B)** Data is represented as mean ± SEM. ^**^*P* < 0.005.

Figure S7 Gating strategy for dengue infection in GFP-DDX3X transfected cells. HEK293T cells transfected with GFP-DDX3X constructs were infected with DENV2 at 5 MOI at 24 h pt. At 24 h pi, cells were fixed and stained with APC-conjugated anti-DENV envelope antibody. Single cell population was gated in FSC-H vs. FSC-A plot and SSC-A vs. FSC-A plot. Live cells were gated for analysis. Dengue-E positive cells were gated in total GFP, GFP^low^ and GFP^high^ respectively.

Figure S8 Overexpression of HA-DDX3X and GFP-DDX3X. HA- or GFP-tagged DDX3X constructs were transfected in HEK-293T cells and at 48 h p.t, cells were fixed and HA-DDX3X transfection samples were stained with HA antibody followed by secondary antibody conjugated with Alexa-488. Nuclei was stained with DAPI.

Figure S9 Overexpression of DDX3X plasmids does not affect cell viability. GFP-tagged DDX3X constructs were transfected in HEK-293T cells and at 24 h p.t, cells were infected with DENV at 5 MOI. **(A–D)** The fixable live-dead viability stain (BD EF-780) was added at 1:1000 dilution and incubated at RT for 10 min and then washed with FACS buffer. Cells were processed by flow cytometry.

Figure S10 IFN-β levels in Huh-7 in DDX3X knockdown and DENV infection. siRNA knockdown of DDX3X was done in Huh-7. Forty-eight hour p.t, cells were either mock or DENV infected at 10 MOI. Twenty-four hour p.i the IFN-β mRNA levels were assessed by real-time PCR. Data is represented as mean ± SEM.
