# Supplementary figures and images for: Dengue Virus Capsid Interacts with DDX3X–A Potential Mechanism for Suppression of Antiviral Functions in Dengue Infection
Source: Front Cell Infect Microbiol. 2018 Jan 17;7:542. doi: 10.3389/fcimb.2017.00542 (PMC5776122; doi:10.3389/fcimb.2017.00542)

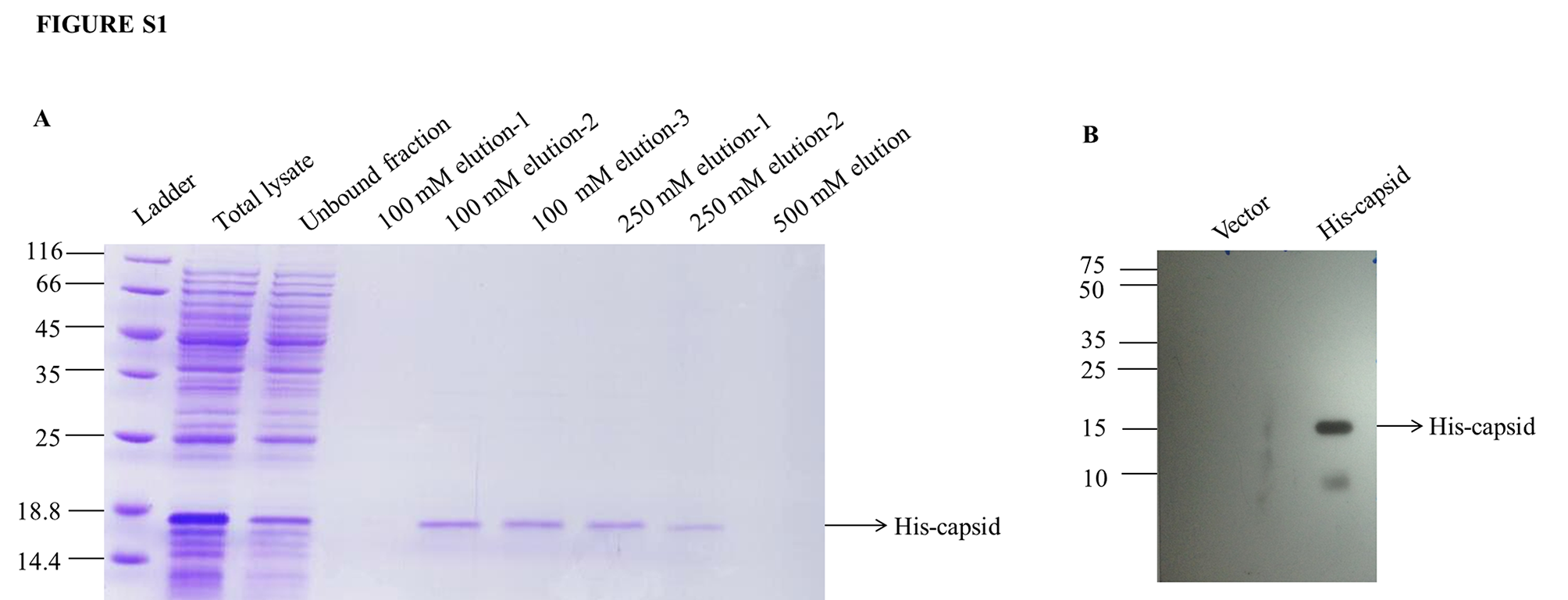

Supplement: Figure S1 — Purification of His-capsid protein. (A) Purification of the His-capsid protein as described in methodology using Ni-NTA beads. (B) Western blot showing purified His-capsid probed using anti-His antibody. [file Image1.TIF]

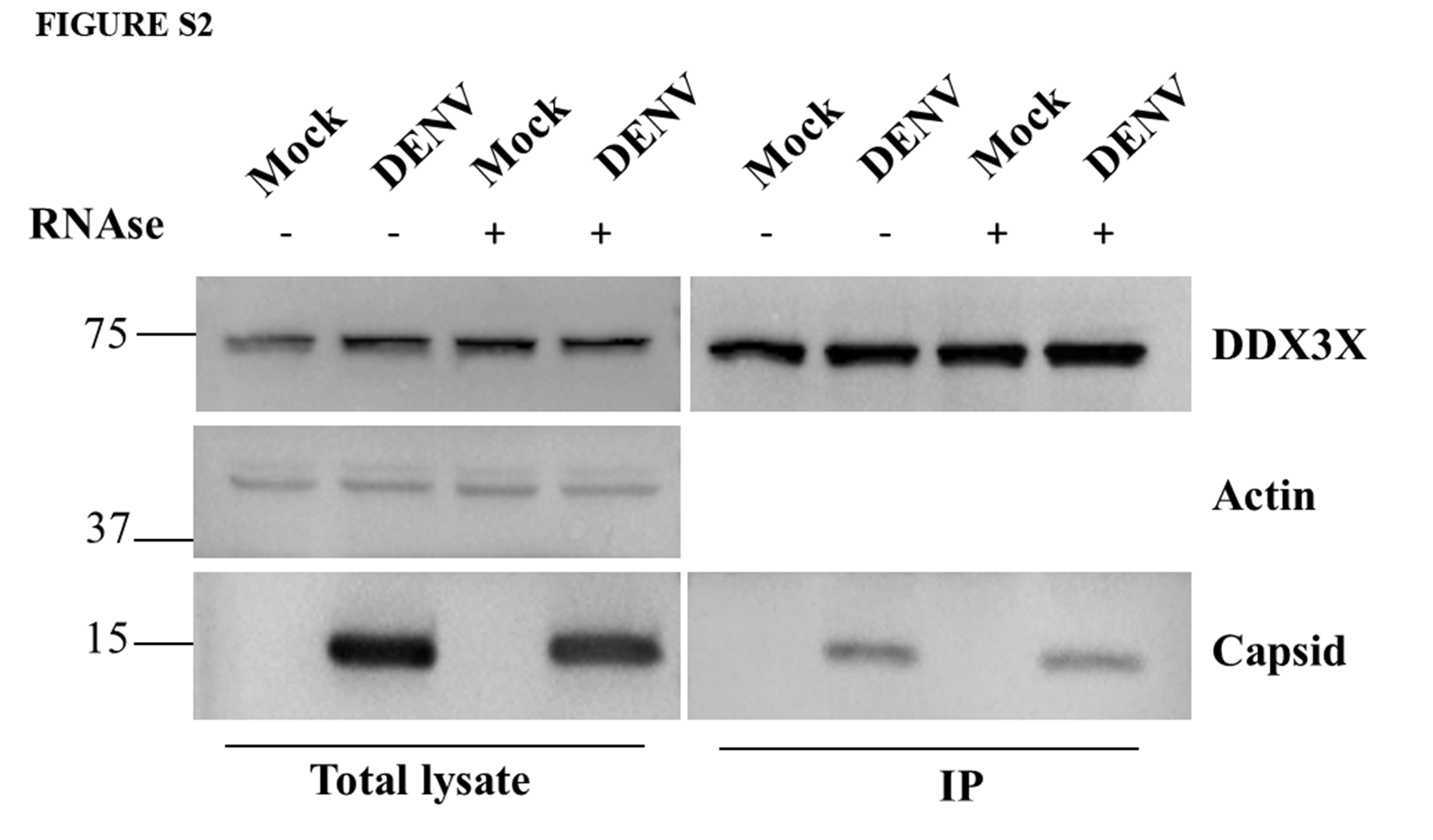

Supplement: Figure S2 — Interaction between capsid and DDX3X is not mediated via RNA. Huh-7 cells were infected with DENV at an MOI of 5. At 24 h pi mock and DENV-infected Huh-7 cell lysates were prepared and treated with RNAse prior to immunoprecipitation. DDX3X was immunoprecipitated from total lysates (untreated) and RNAse-treated lysates using DDX3X antibody at 4°C. Total lysate and immunoprecipitated proteins were detected by western blots. [file Image2.TIF]

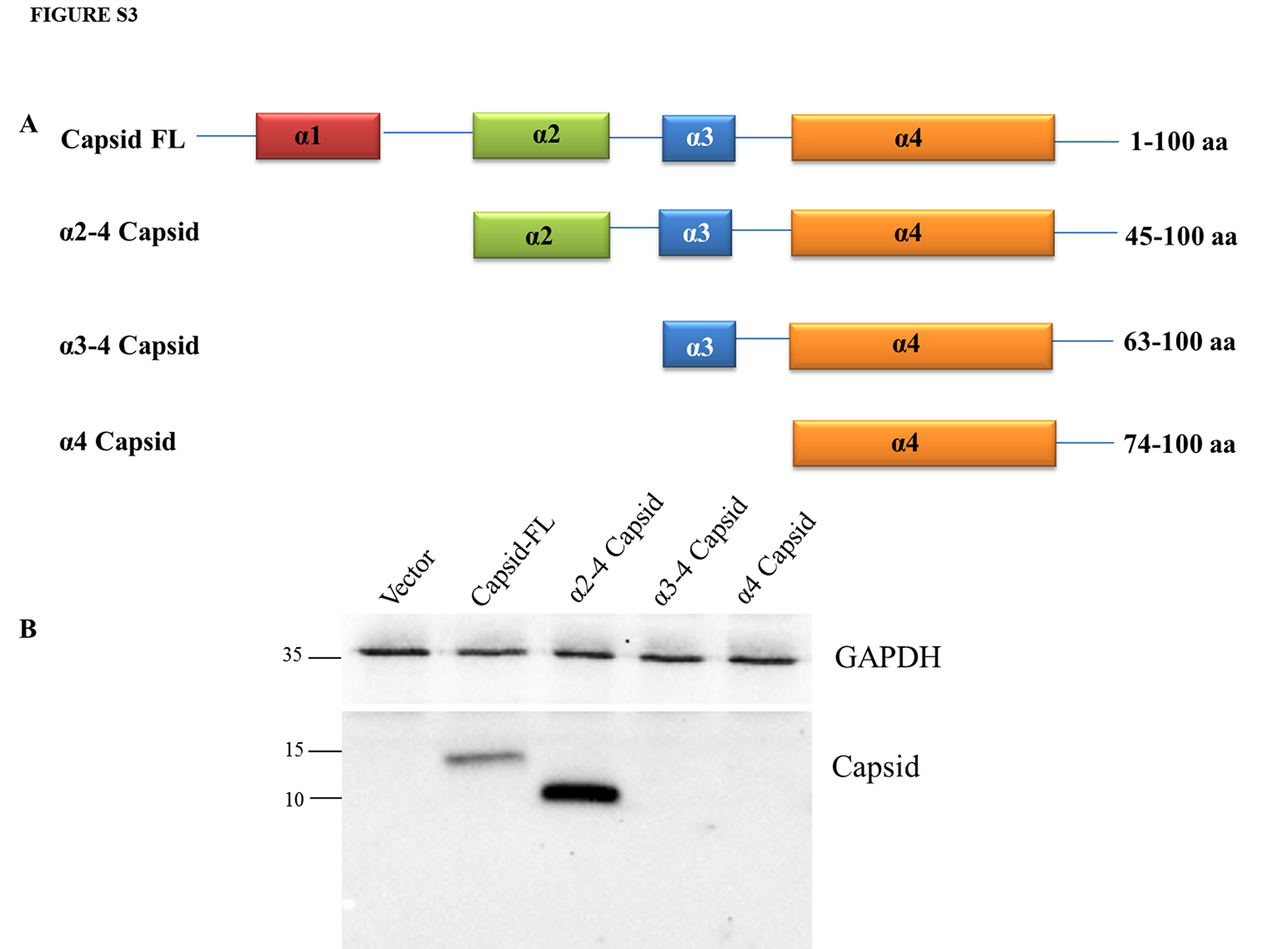

Supplement: Figure S3 — Deletion constructs of capsid. (A) Graphical representation of His-capsid deletion constructs. (B) HEK293T cells were transfected with plasmids encoding various capsid constructs. Total lysates were prepared at 24 h p.t. and expression of recombinant His-tagged proteins were analyzed by western blotting with capsid antibody. GAPDH levels are shown as loading controls. [file Image3.TIF]

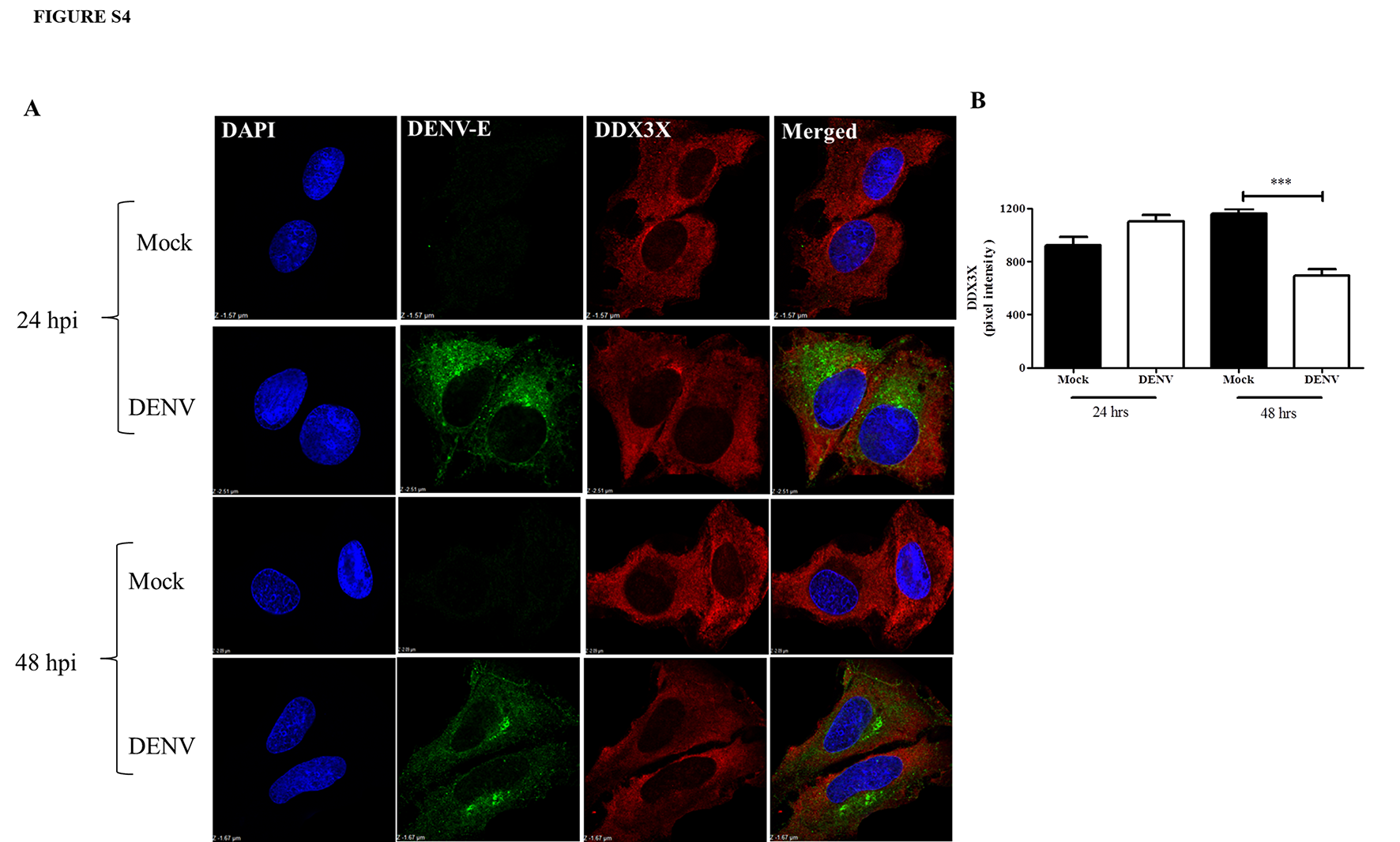

Supplement: Figure S4 — DENV infection downregulates DDX3X in Huh-7 cells at later stages of infection. (A) Huh-7 cells were infected at 1 MOI and fixed at 24 and 48 h pi. Cells were stained for DDX3X (red), dengue envelope (green) and nuclei with DAPI (blue). (B) The fluorescence intensity of DDX3X staining was quantitated using Fluoview software. Quantitation of DDX3X fluorescence intensity (AU) at 24 and 48 h pi. (n = 15) ***P < 0.0005. [file Image4.TIF]

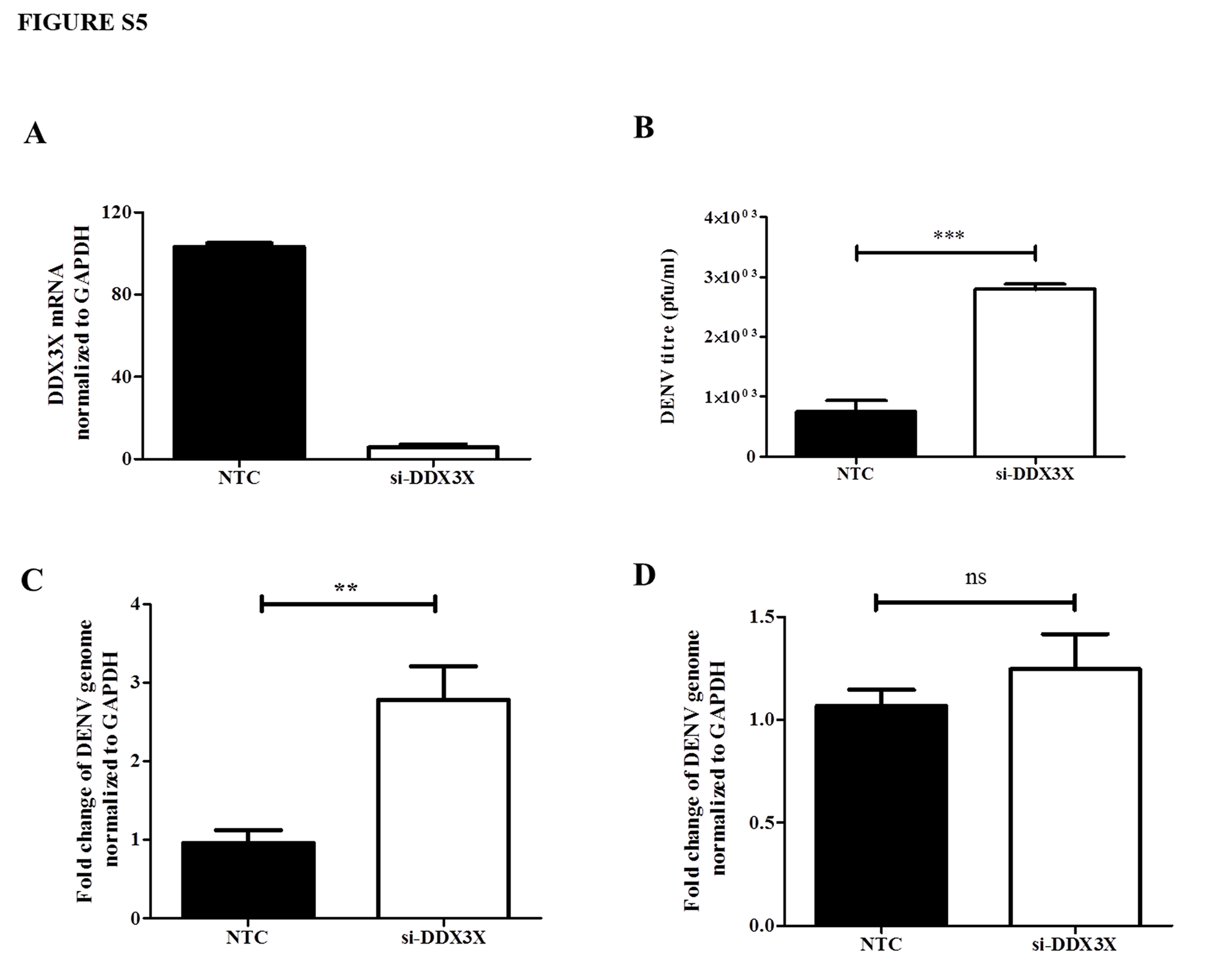

Supplement: Figure S5 — DDX3X downregulation enhances DENV infection in A549 cells. (A) Cells were transfected with si-NTC or si-DDX3X and at 48 h p.t. knock-down efficiency was measured by real-time PCR from total RNA. (B) Cells transfected with si-NTC or si-DDX3X were infected with DENV at 3 MOI at 48 h p.t. Viral titres were estimated at 24 h pi by plaque assay and viral genome levels were analyzed by real-time and normalized to GAPDH (C) Data represents three independent experiments performed with triplicates samples (n = 9). (D) Viral entry was assessed in DDX3X knockdown cells. Cells were collected after 1 h of virus adsorption and viral genome levels was analyzed by real-time PCR. Data presented are from two independent experiments performed with three replicates each (n = 6). Data is represented as mean ±SEM. **P < 0.005 and ***P < 0.0005, ns = not significant. [file Image5.TIF]

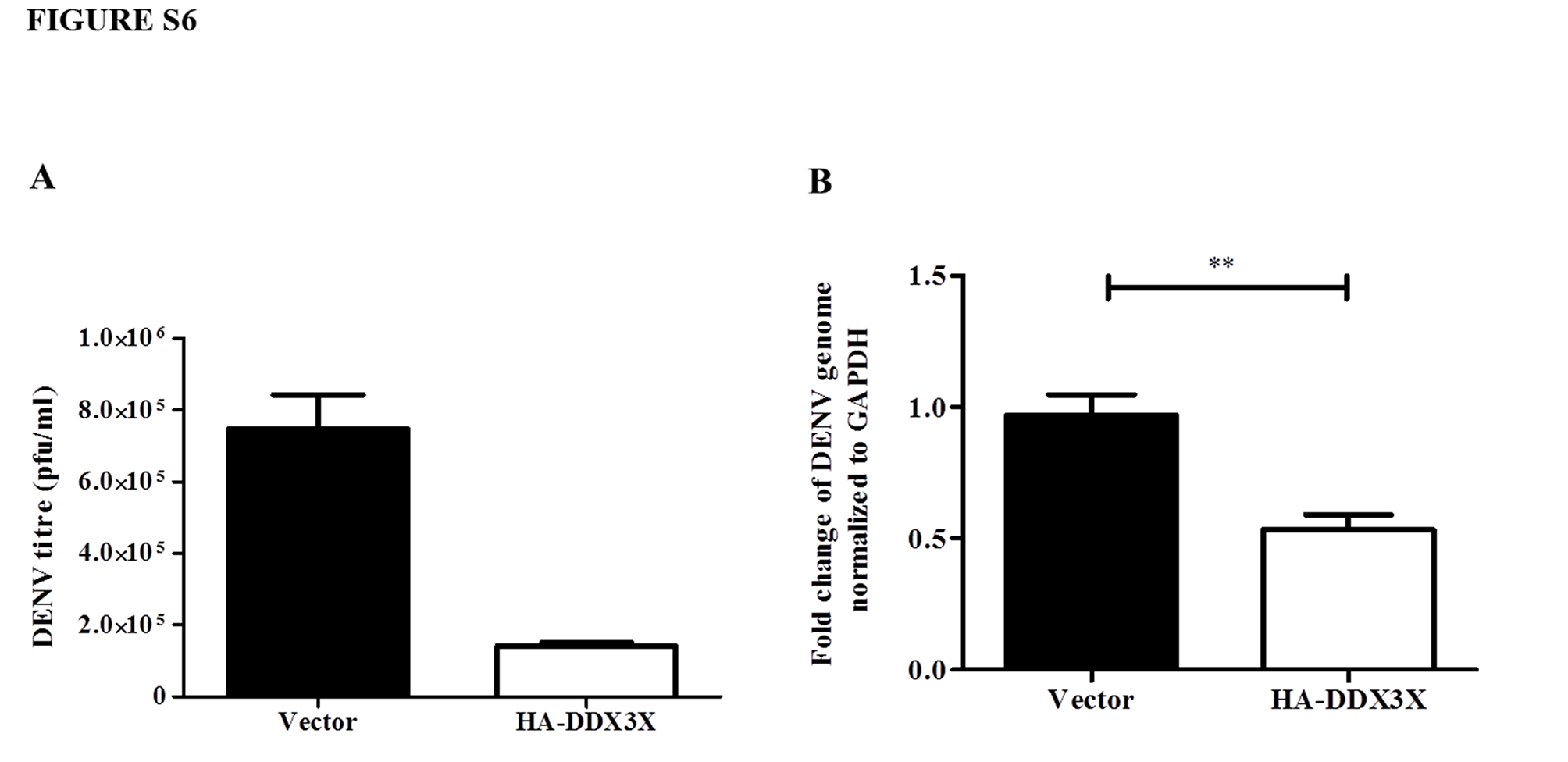

Supplement: Figure S6 — Overexpression of HA-DDX3X in Huh-7 cells inhibits virus replication. HA-DDX3X was transfected in Huh-7 cells, and at 24 h p.t, cells were infected with DENV at 3 MOI and viral titers were measured by plaque assay at 24 h pi (A) DENV viral genome levels were estimated by real-time PCR. Data is from three independent experiments performed in triplicates (n = 9). (B) Data is represented as mean ± SEM. **P < 0.005. [file Image6.TIF]

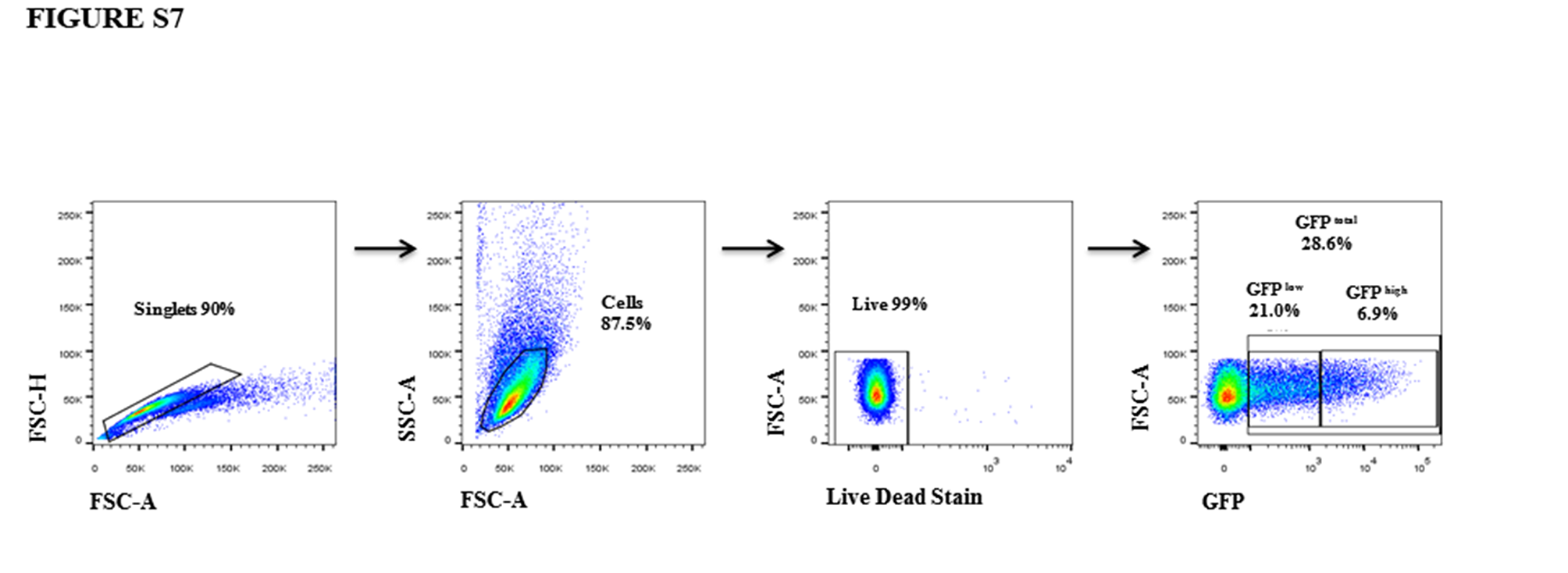

Supplement: Figure S7 — Gating strategy for dengue infection in GFP-DDX3X transfected cells. HEK293T cells transfected with GFP-DDX3X constructs were infected with DENV2 at 5 MOI at 24 h pt. At 24 h pi, cells were fixed and stained with APC-conjugated anti-DENV envelope antibody. Single cell population was gated in FSC-H vs. FSC-A plot and SSC-A vs. FSC-A plot. Live cells were gated for analysis. Dengue-E positive cells were gated in total GFP, GFPlow and GFPhigh respectively. [file Image7.TIF]

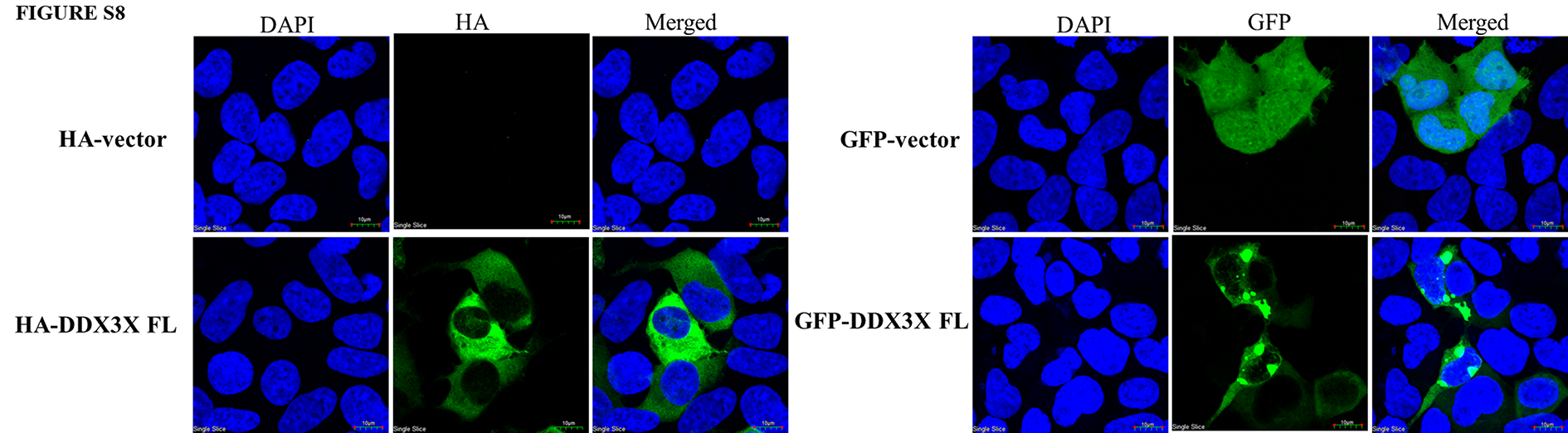

Supplement: Figure S8 — Overexpression of HA-DDX3X and GFP-DDX3X. HA- or GFP-tagged DDX3X constructs were transfected in HEK-293T cells and at 48 h p.t, cells were fixed and HA-DDX3X transfection samples were stained with HA antibody followed by secondary antibody conjugated with Alexa-488. Nuclei was stained with DAPI. [file Image8.TIF]

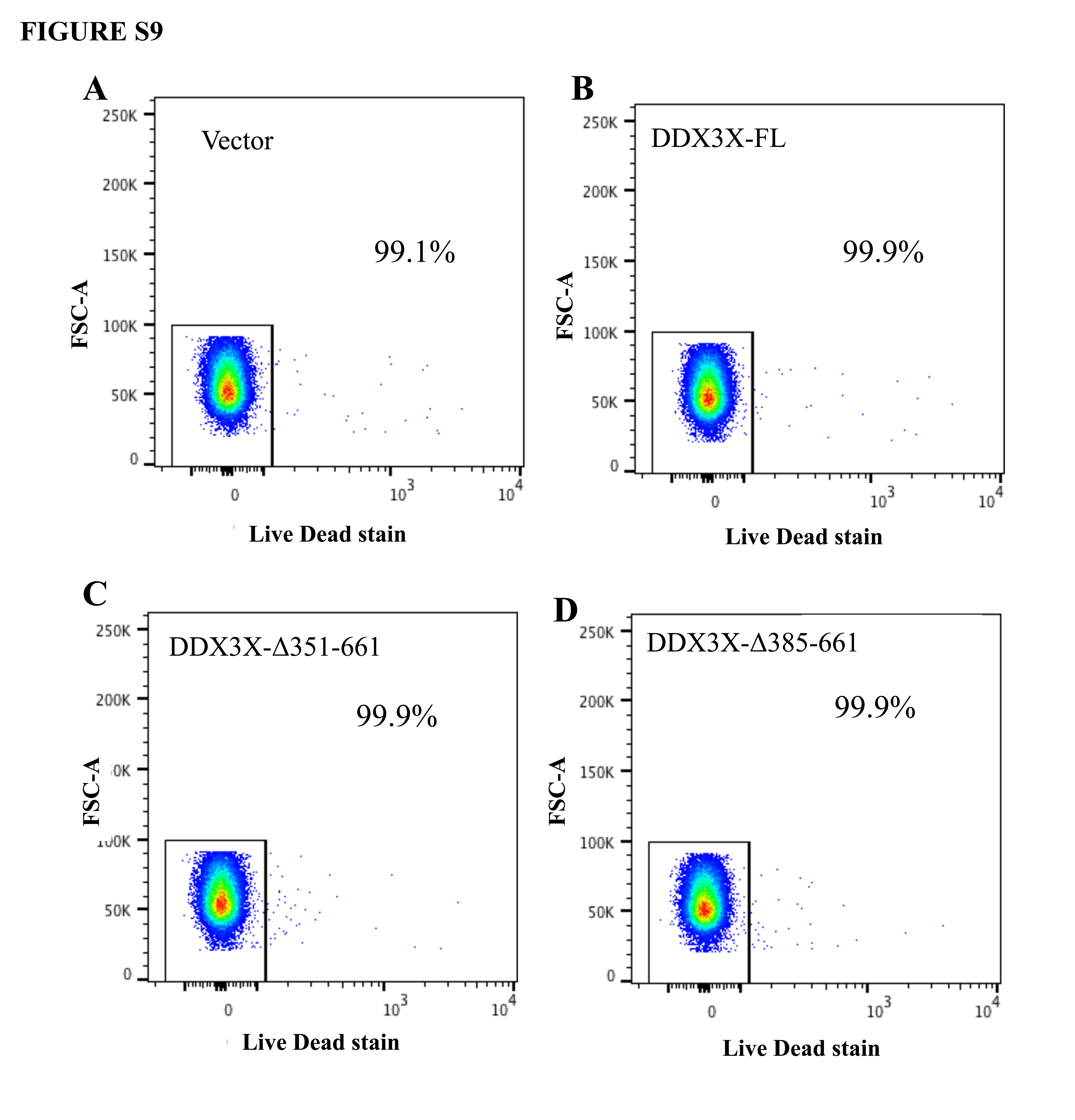

Supplement: Figure S9 — Overexpression of DDX3X plasmids does not affect cell viability. GFP-tagged DDX3X constructs were transfected in HEK-293T cells and at 24 h p.t, cells were infected with DENV at 5 MOI. (A–D) The fixable live-dead viability stain (BD EF-780) was added at 1:1000 dilution and incubated at RT for 10 min and then washed with FACS buffer. Cells were processed by flow cytometry. [file Image9.TIF]

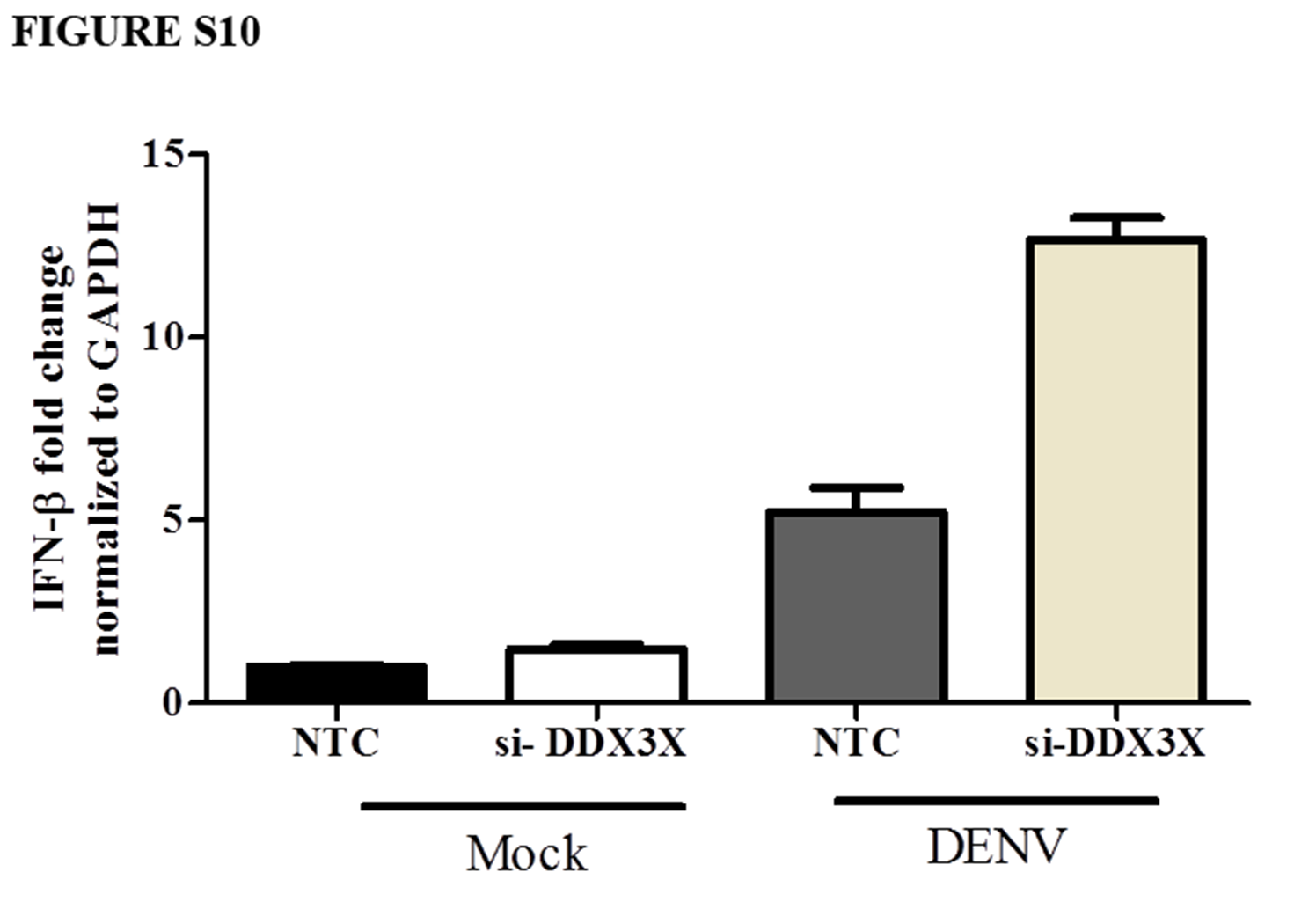

Supplement: Figure S10 — IFN-β levels in Huh-7 in DDX3X knockdown and DENV infection. siRNA knockdown of DDX3X was done in Huh-7. Forty-eight hour p.t, cells were either mock or DENV infected at 10 MOI. Twenty-four hour p.i the IFN-β mRNA levels were assessed by real-time PCR. Data is represented as mean ± SEM. [file Image10.TIF]
